# Temperature and CO_2_ concentration in honey bee hives exhibit circadian rhythms

**DOI:** 10.1038/s41598-025-03614-3

**Published:** 2025-07-01

**Authors:** W. G. Meikle, M. Weiss

**Affiliations:** Carl Hayden Bee Research Center, USDA-ARS, 2000 E. Allen Rd, Tucson, AZ 85719 USA

**Keywords:** Honey bee colonies, Superorganism, Continuous monitoring, Cold storage, Colony-level behavior, Periodogram analysis, Cosinor analysis, Ecology, Behavioural ecology

## Abstract

Worker honey bees exhibit circadian rhythms with respect to locomotor activity but circadian analyses have seldom been applied to colony-level behavior. Circadian rhythms have been defined as having three main characteristics: a period of approximately 24 h maintained in the absence of external cues; a period maintained over a range of temperatures; and a phase fixed by external cues from the environment. In this study honey bee colonies were subjected to two kinds of light regimes at 5 °C in a cold storage unit (CSU): (1) constant darkness; and (2) after 6–12 d constant darkness, 12 h light exposure from 6PM to 6AM (i.e. a phase approximately 12 h offset from ambient conditions). Periodogram analyses of data from the 1st light regime showed that temperature and CO_2_ concentration had stable 24 h periods after 20 d, as was observed for colonies in warmer temperatures in outside conditions. Period strength of temperature decreased over time in the CSU but not CO_2_. Cosinor analyses of data from the 2nd light regime showed a temperature phase change of about 9 h 37 min between the end of the CSU period, after 28–33 d in light regime, and after 7–12 d in outdoor conditions in the post-CSU period. The same comparison for CO_2_ concentration showed a phase change of about 11 h 55 min. These data indicated honey bee colonies produced circadian rhythms in hive temperature and CO_2_ concentration with periods both present in the absence of external cues, and with phases that can be driven by light. Rhythms associated with CO_2_ concentration changed with respect to light treatment more than rhythms associated with hive temperature. Based on data from longer-term (60 d) experiments, daily rhythm phases and day lengths differed significantly between hive temperature and CO_2_ after 15 d in the 12 l:12D light regime, and remained so in outdoor conditions.

**Supplementary Information:**

The online version contains supplementary material available at 10.1038/s41598-025-03614-3.

## Introduction

Many organisms, including animals and plants, exhibit circadian rhythms in various behavioral and physiological processes^1^. Worker honey bees exhibit circadian rhythms in their locomotor activity^2–5^. Locomotor activities of individuals in a laboratory setting, the typical response variable for circadian studies, are comparatively easy to measure by placing individual bees in a controlled environment and observing how activity changes with respect to changes in light, temperature and other factors^2,3^. However, honey bees do not exist naturally as single individuals; all are members of colonies considered by some to be “superorganisms”^6^ and those colonies exhibit behavior that is not simply the summed behaviors of the colony members. Circadian rhythm characteristics of colony-level behaviors have seldom been studied.

Thermoregulation and control of CO_2_ concentration within the hive are two examples of emergent properties of bee colonies with little correspondence to the behaviors of isolated individual bees. Individual activities contribute in different ways to colony-level activities. Food collection, for example, involves foraging as well as the collection of the material from the foragers, the passing of the material among other workers and consumption or storage of material in the comb^7^. Both thermoregulation and CO_2_ concentration control require comparatively sophisticated colony-level behavior in order to monitor the current situation and to take action to improve the situation if needed, but are themselves very different. In the presence of brood, colonies tend to maintain temperatures of 34–35 °C within the cluster^8^ with a small temperature range (usually < 1 °C) irrespective of the ambient temperature. This thermoregulation is an example of a collective behavior requiring some workers to provide heat when outside temperatures are low, or to provide water for evaporative cooling when outside temperatures are high, and other workers to play various roles in fanning, insulating the cluster, or leaving the hive (when temperatures are very high). In this case the difference between colony and individual behavior is clear: outside the colony an isolated bee typically behaves as a poikilothermic exotherm, whereas a colony behaves as a homeothermic endotherm, generating and maintaining a desirable temperature range. To control CO_2_ concentration honey bee workers have fewer options: (1) fanning or not fanning; and (2) remaining within the hive or exiting the hive to avoid buildup of gases within the hive. All colony members constantly produce CO_2_, but some activities, including fanning and increasing body temperature (as part of their thermoregulation behavior) change the rate of CO_2_ production.

Circadian rhythms have been defined as having three main characteristics: (1) rhythms with periods of approximately 24 h that are maintained in the absence of external cues; (2) the period is maintained over a range of temperatures; and (3) the phase of the rhythms is fixed by external cues from the environment^9^. Circadian rhythms in honey bee locomotor activity can be entrained by light and temperature^10^ as well as by substrate-borne vibrations generated by forager activity, and by volatiles, including possibly CO_2_^11,12^.

Honey bee locomotor activity exhibits circadian rhythmicity, but do colony-level behaviors exhibit it as well? Honey bee colonies in typical outdoor settings have been found to exhibit strong 24 h cycles in both temperature^13^ and CO_2_ concentration^14^, suggesting it is possible that they do. Thermoregulation in a bee hive is driven by ambient conditions^6^, and colonies thermoregulate even in the absence of brood^14^. How intensely bee colonies thermoregulate is a function of a number of factors, including subspecies^15^, genetic diversity within the colony^16^, and pesticide exposure^17^. Temperature data are also affected by colony size and sensor location within the hive^14^. A sensor in or near the mass of bees (the “cluster”) at the core of the colony is affected less by outside conditions than one further from cluster, although distance to cluster can change as clusters change size and move during the year^18^. Hive CO_2_ concentration has likewise been found affected by different factors, including pesticide exposure^17^ and hive design^14^. While daily changes in ambient temperature can exceed by an order of magnitude those observed within the hive, daily changes in ambient CO_2_ concentration are exceeded by several orders of magnitude those observed in the hive^14^. If CO_2_ concentration can be considered as a “surrogate” for foraging activity^1^, which is at least partly a function of light and temperature, then CO_2_ concentration may also be a surrogate for a general concerted colony activity.

In this study we examined whether (1) thermoregulation and hive CO_2_ concentration exhibit 24 h periods in the absence of daily temperature and light cues; and (2) the phases of the temperature and CO_2_ concentration data can be altered by altering an external cue, the light regime, in cold storage. First, periodogram analyses were conducted on data from bee colonies kept in constant darkness and a constant low temperature in a cold storage unit (hereafter “CSU”) to determine whether the data from those colonies maintained a 24 h period over three weeks. Second, periodogram results from the CSU were compared to data from the same colonies in outdoor conditions after the CSU treatment, as well as from colonies maintained in outdoor apiaries during the CSU treatment, to examine whether signal periods were affected by ambient temperature. Third, a cosinor analysis was conducted on data from colonies kept in constant low temperatures in the CSU but with a daily light regime approximately 12 h offset from outdoor conditions to determine whether that light phase offset could change the phase of the temperature and CO_2_ data. Five separate experiments were conducted, and each experiment involved a unique set of colonies (Supplementary Information Table S1).

## Results

Honey bee colony sizes in adult bee mass and in the surface area of the brood are provided (Supplementary Information Table S2). Raw temperature data showed that in-hive temperatures changed very differently over time, depending on whether the colonies were inside the CSU or outside, likely due to reduced brood production by colonies in the CSU^19^. Ambient temperature fluctuation within the CSU across all five experiments, due to equipment and to the colonies themselves, was 4.0 ± 0.3 °C (Supplementary Information Figure S1). An examination of a 3-d subset, taken during the latter part of the storage period, from a hive kept in the CSU and one kept outside did show some daily differences; the ambient temperature data from the CSU did not show strong daily patterns (Supplementary Information Figure S2). Raw CO_2_ data showed very different patterns between colonies kept in the CSU and those kept outside the CSU as well as between the two years of that study (Supplementary Information Figure S3), likely due to the different sizes of the colonies and to the presence of entrance screens in one experiment but not the other^19^. A sample 3-d subset illustrated the different patterns between treatment groups and between years of the study (Supplementary Information Figure S4).

### Strength of 24 h periods in temperature and CO_2_ data in different temperature regimes.

**Fig. 1:**
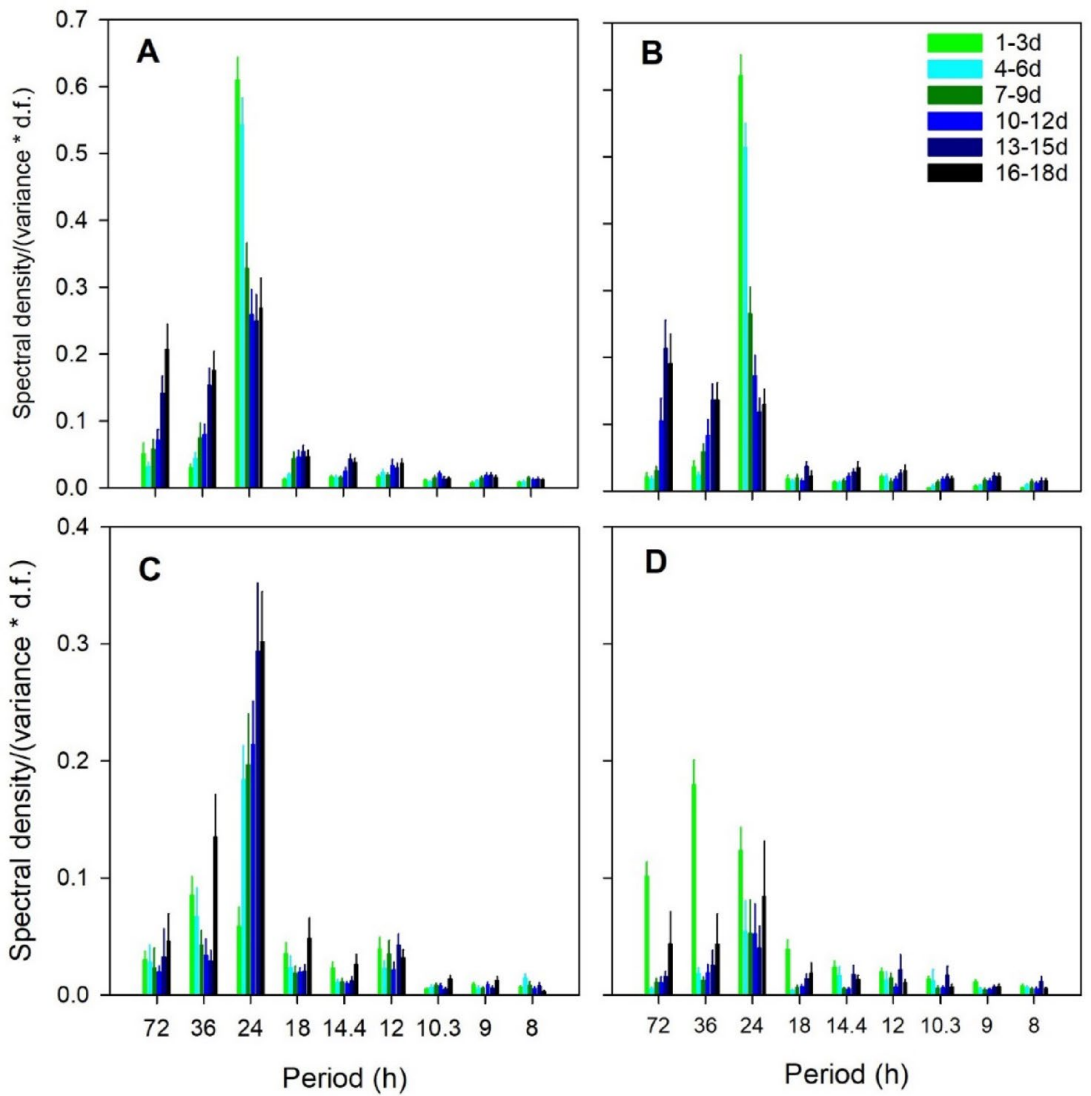
Proportion sums of squares (average ± s.e.), calculated as the spectral density divided by the product of the variance and the degrees of freedom, for periods ≥ 8 h for detrended temperature and CO_2_ concentration data (calculated using a 48 h moving average). Shown are periodogram results for each of six consecutive 3-d data subsets. (**A**) Temperature in Fall 2020 experiment; (**B**) Temperature in Fall 2021 experiment; (**C**) CO_2_ concentration in Fall 2020 experiment; (**D**) CO_2_ concentration in Fall 2021 experiment.

 Periodograms derived from raw in-hive temperature data (Supplementary Information Figure S5) and from detrended data (Supplementary Information Figure S6) showed that 24 h signals were typically the most important (signals at 48 h and 72 h may be harmonics of the 24 h signal, and their contribution was reduced by using a 48 h running average to detrend the raw data). Air was forced into the CSU for 4–5 min each hour, but the signal for the 1 h period was weak in the ambient data and negligible for the in-hive data, indicating the effects of air exchange on temperature were comparatively weak. The magnitude of the 24 h signal significantly decreased over time, suggesting that the naturally occurring 24 h cycle of ambient light and temperature played an important role in maintaining that signal (Fig. 1). For temperature data from colonies in the CSU, the strength of the 24 h signal in subsets 1, 2 and 3 decreased significantly over time, while the signals in subsets 4, 5 and 6 were not different from each other. Data from colonies in ambient conditions outside the CSU did not show a consistent pattern (Supplementary Information Table S3). The reason for the changes in the strength of the 24 h signal among hives kept outside is unclear, but those colonies were subjected to much higher daily ambient temperature fluctuations of 20–30°C^20^ than colonies in the CSU. Nevertheless, the 24 h signal remained the strongest signal.

Periodogram signals derived from raw in-hive CO_2_ data (Supplementary Information Figure S7) and from detrended data (Supplementary Information Figure S8) showed that, as with the temperature data, 24 h signals were typically the most important signals. In contrast to the temperature data, CO_2_ data from colonies within the CSU did not show any significant changes in the magnitude of the 24 h signal over time (Supplementary Information Table S3). Colonies kept outside the CSU showed some changes (subsets 3 and 6 were low compared to other periods) but no consistent pattern. This result suggests that the temperature and CO_2_ cycles are not necessarily driven by the same cues.

**Fig. 2:**
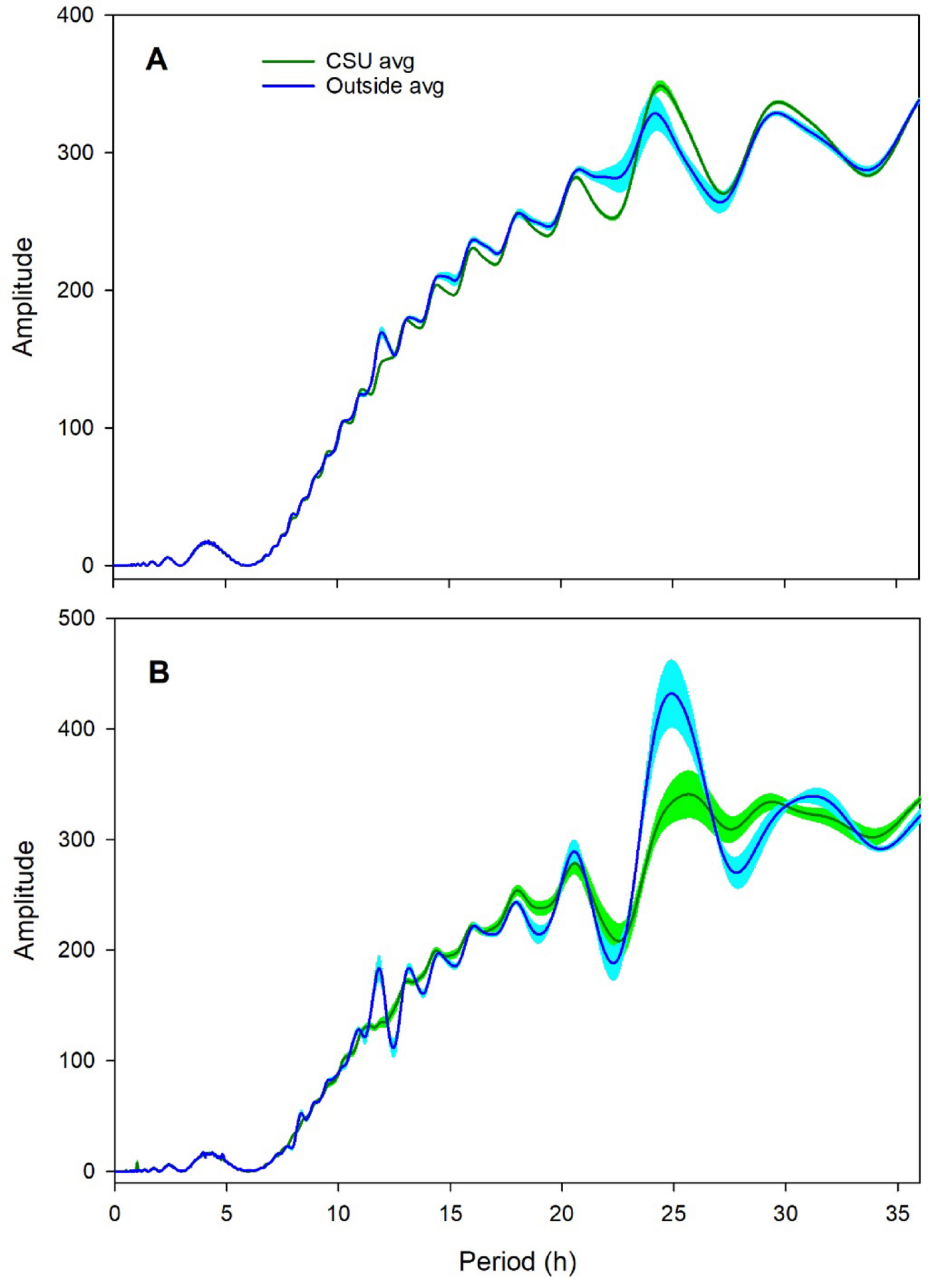
Lomb-Scargle periodogram averages for 6 d raw data from the Summer 2023 experiments for colonies kept under total darkness (“CSU avg”) and colonies kept outside during the same period (“Outside avg”). (**A**) Hive temperature; and (**B**) Hive CO_2_ concentration. Lighter colors show standard errors for each line.

More precise estimates of daily period were obtained from Summer 2023 experiments, when data were collected every 5 min., using Lomb-Scargle periodogram analyses. Those analyses showed that temperature had an average period of 25.4 ± 0.5 h for colonies in 6 d total darkness in the CSU, compared to 27.3 ± 0.5 h for the same colonies outside, while CO_2_ concentration had an average period of 27.5 ± 0.6 h for colonies in 6 d total darkness in the CSU, compared to 25.5 ± 0.4 h for colonies outside (Fig. 2). A Lomb-Scargle periodogram analysis of temperature and CO_2_ concentration data after 7–12 d total darkness in the Winter 2023 and Winter 2024 experiments is provided (Supplementary Information Figure S9).

### Changing the phases of the temperature and CO_2_ cycles using light exposure

**Fig. 3:**
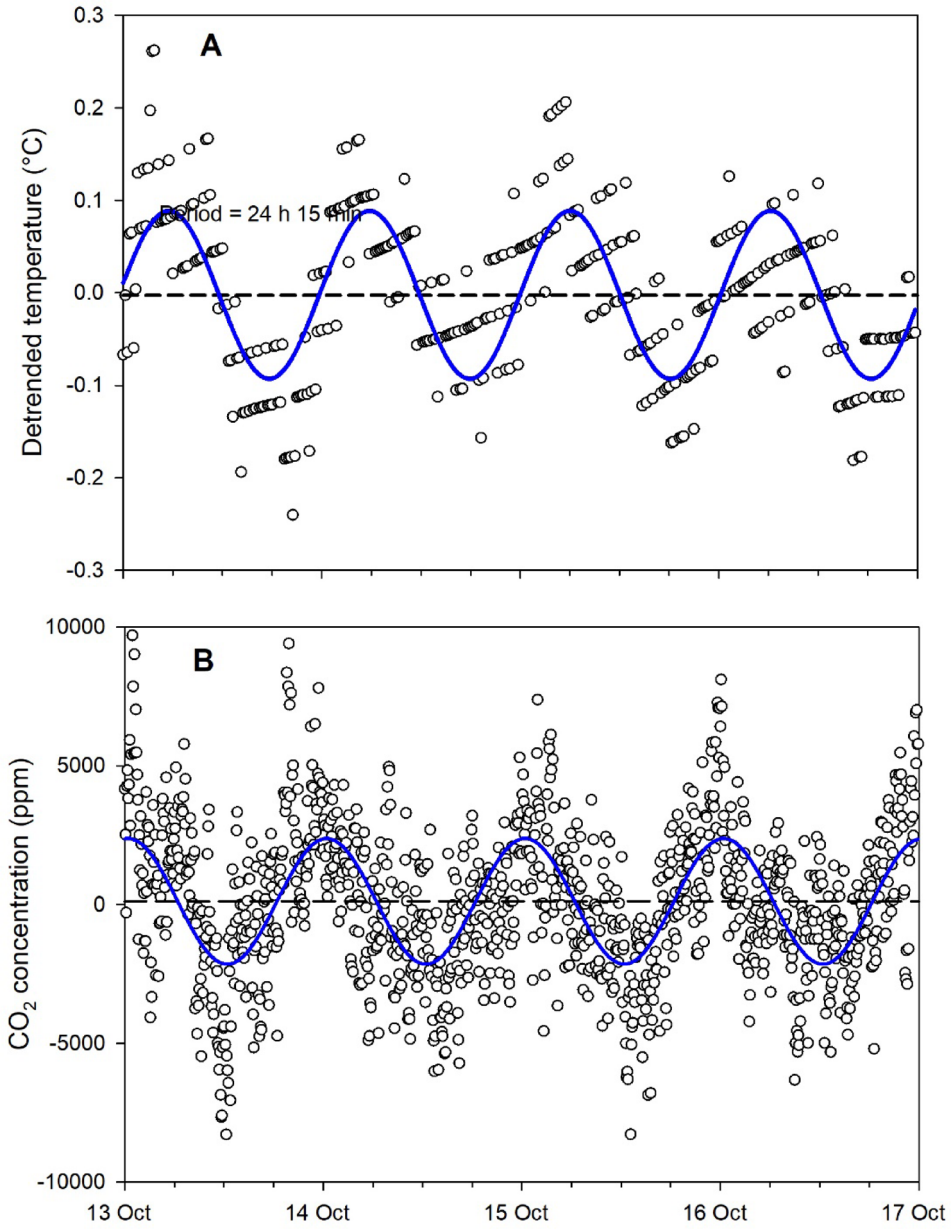
Sample detrended sensor data from a hive in the Fall 2021 experiment with curves fit from cosinor analysis. (**A**) Temperature; (**B**) CO_2_ concentration. Horizontal dashed line shows the mesor (Midline Statistic Of Rhythm, a rhythm-adjusted mean).

**Fig. 4:**
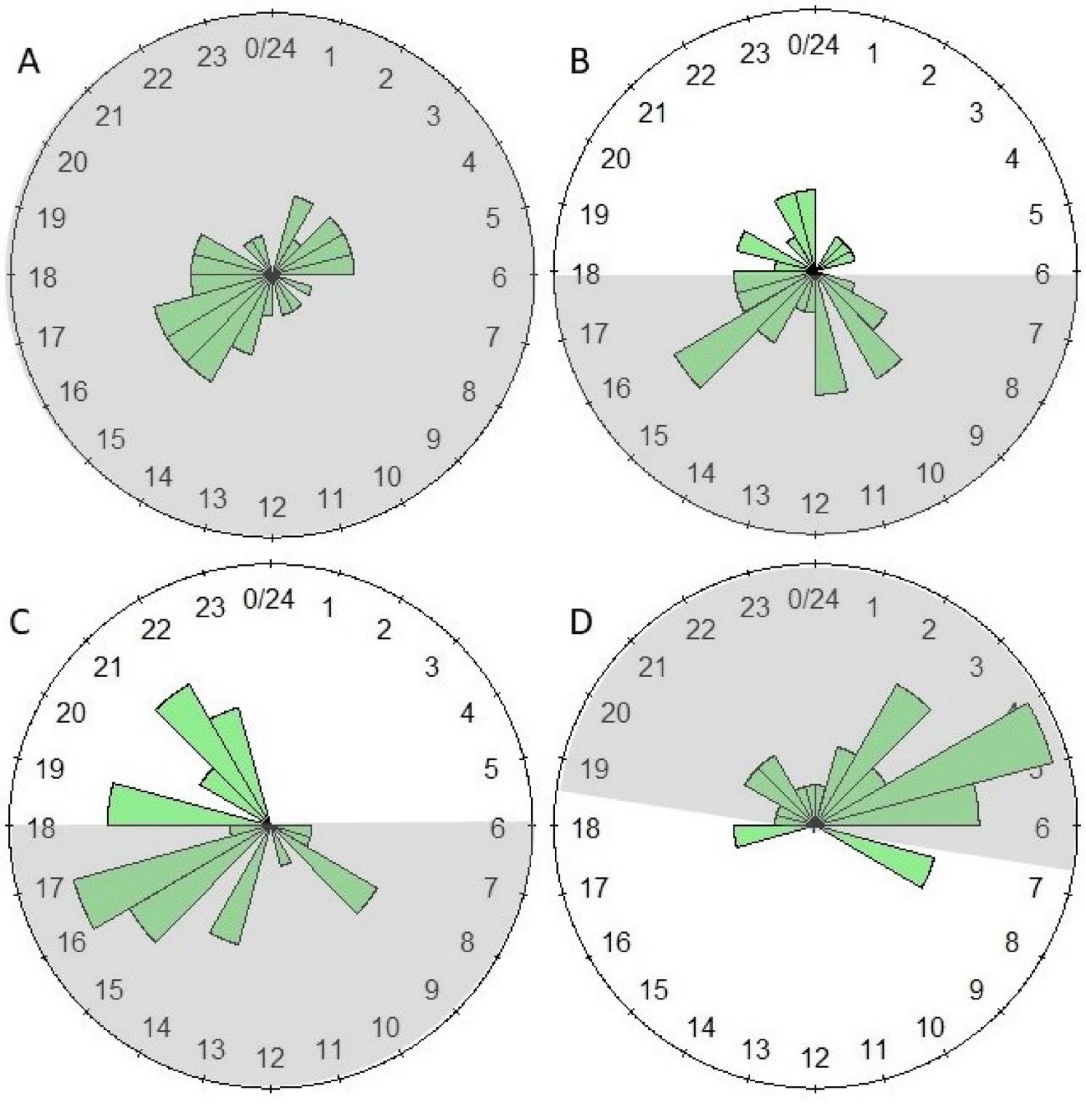
Rose diagram of the phase distribution resulting from a cosinor analysis of within-hive detrended temperature data from the Winter 2023 and 2024 experiments. Shown are the phase distribution binned by hour for 16 hives across two 3-d subsets (= 32 data points) for each graph. Gray shading shows dark periods (from dusk to dawn for hives outside). (**A**) Days 7–12 in CSU under total darkness; (**B**) Days 25–30 under a 12 l:12D light regime, with light starting at 6:00 PM and ending at 5:59 AM; (**C**) Days 40–45 under the same 12 l:12D regime; and (**D**) Days 7–12 post-CSU under ambient light and temperature conditions.

**Fig. 5:**
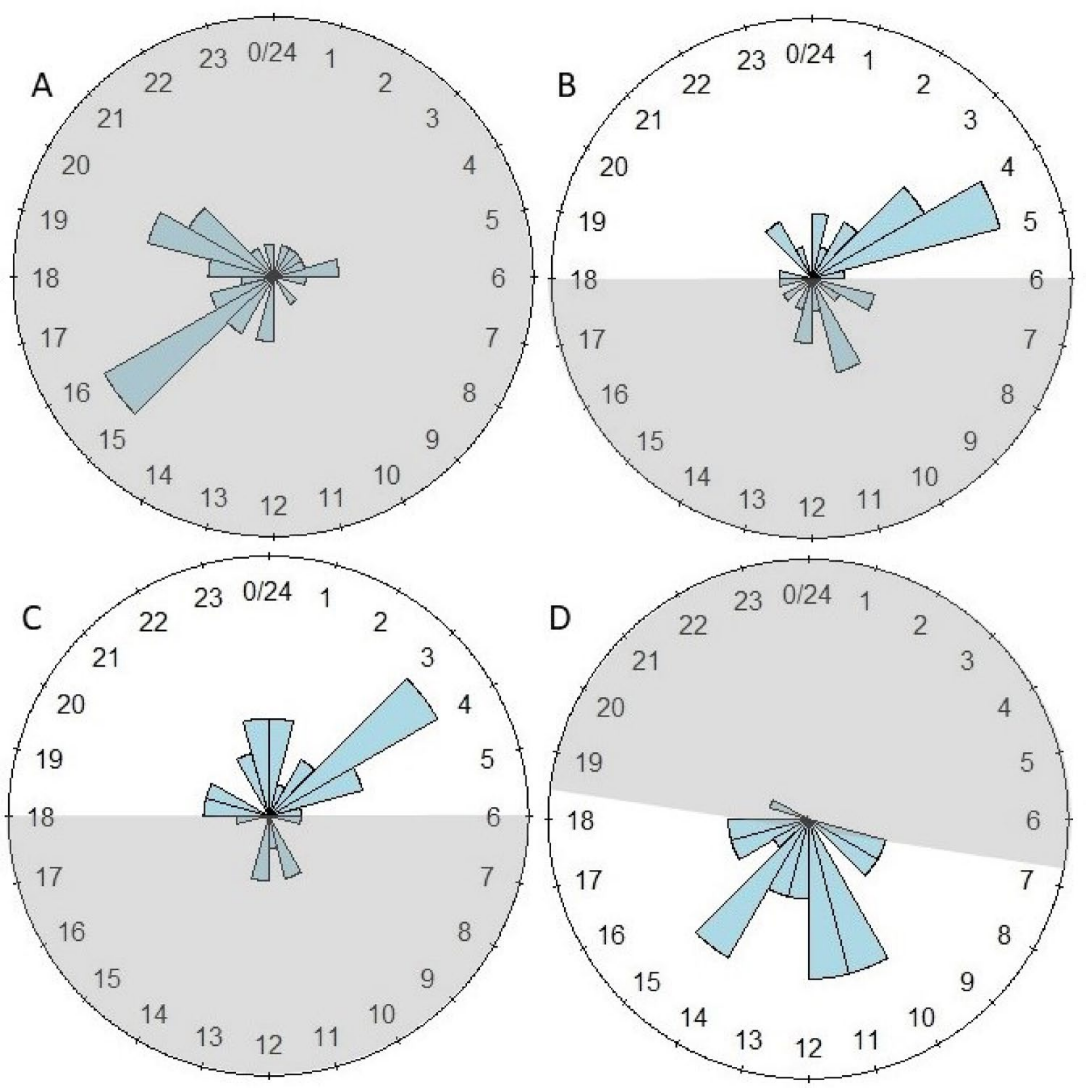
Rose diagram of the phase distribution resulting from a cosinor analysis of within-hive detrended CO_2_ data from the Winter 2023 and 2024 experiments. Shown are the phase distribution binned by hour for 16 hives across two 3-d subsets (= 32 data points) for each graph. Gray shading shows dark periods (from dusk to dawn for hives outside). (**A**) Days 7–12 in CSU under total darkness; (**B**) Days 25–30 under a 12 l:12D light regime, with light starting at 6:00 PM and ending at 5:59 AM; (**C**) Days 40–45 under the same 12 l:12D regime; and (**D**) Days 7–12 post-CSU under ambient light and temperature conditions.

 The mean and median values of the phases are provided for all experiments, as well as the fits of the phase distributions to a von Mises distribution (Supplementary Information Tables S4, S5 and S6). An example of cosinor fits to sample temperature and CO_2_ data from the same hive during the same interval is provided (Fig. 3). With respect to the Summer 2023 data, two of the seven temperature intervals (3 intervals each for hives in the CSU and outside, in addition to the post-CSU interval) and four of the seven CO_2_ intervals were significantly different from the von Mises distribution. However, with respect to the Winter 2023 and Winter 2024 data only two intervals (25–30 d CSU and post CSU temperature data) were significantly different from the von Mises distribution. Results of pairwise comparisons across all experiments are provided (Table 1, and Supplementary Information Tables S7, S8 and S9). Rose diagrams with 1 h bins of the temperature and CO_2_ data for the Fall 2020 and Fall 2021 experiments (Supplementary Information Figures S10 and S11), the Summer 2023 experiments (Supplementary Information Figures S12 and S13), and the Winter 2023 and 2024 experiments are provided (Figs. 4 and 5). Caution should be exercised in the interpretation in this analysis of the Summer 2023 and Winter 2023 and 2024 phase data; the error term in the results from the Watson two-sample tests was likely underestimated, because data were pooled for those analyses within each interval (two 3-d subsets) and across hives (16 hives) to generate a large enough sample size for comparison. The independence of data from different 3-d subsets from the same hive would not have been the same as the independence of data from different hives.

**Table 1:** Contrast values (Group 1 – Group 2) for comparisons among periods within sites and sites within period for both winter experiments (2023-24 and 2024-25).

Parameter	Group 1		Group 2		Contrast value	Watson	*P*
	Site	Interval	Site	Interval			
Temperature	CSU	7–12 d	CSU	25–30 d	5.920	0.044	> 0.1
	CSU	7–12 d	CSU	40–45 d	0.514	0.120	> 0.1
	CSU	7–12 d	out	post	**3.032**	**0.479**	**< 0.001**
	CSU	25–30 d	CSU	40–45 d	0.877	0.073	> 0.1
	CSU	25–30 d	out	post	**3.395**	**0.632**	**< 0.001**
	CSU	40–45 d	out	post	**2.518**	**0.880**	**< 0.001**
CO_2_	CSU	7–12 d	CSU	25–30 d	**3.409**	**0.372**	**< 0.01**
	CSU	7–12 d	CSU	40–45 d	**4.157**	**0.374**	**< 0.01**
	CSU	7–12 d	out	post	**0.997**	**0.432**	**< 0.001**
	CSU	25–30 d	CSU	40–45 d	0.748	0.097	> 0.1
	CSU	25–30 d	out	post	**3.871**	**0.521**	**< 0.001**
	CSU	40–45 d	out	post	**3.123**	**0.760**	**< 0.001**

“Site” indicates whether the data are from colonies within the CSU (“CSU”) or outside in ambient conditions (“out”). “Interval” indicates the 6-d interval, with each interval consisting of two sequential 3-d subsets; during interval “7–12 d” colonies were in total darkness; during intervals “25–30 d” and “40–45 d” colonies were subject to a 12 l:12D light regime; and during interval “post” colonies were in ambient conditions outside the CSU. “Watson” indicates the value of the Watson’s test for homogeneity on two samples of circular data, and “P” refers to the probability of the value. *N* = 32 for each group within the CSU; *N* = 30 for the post-CSU temperature group and *N* = 26 for the post-CSU CO_2_ group (see text for details). Significant contrasts are in bold.

**Fig. 6:**
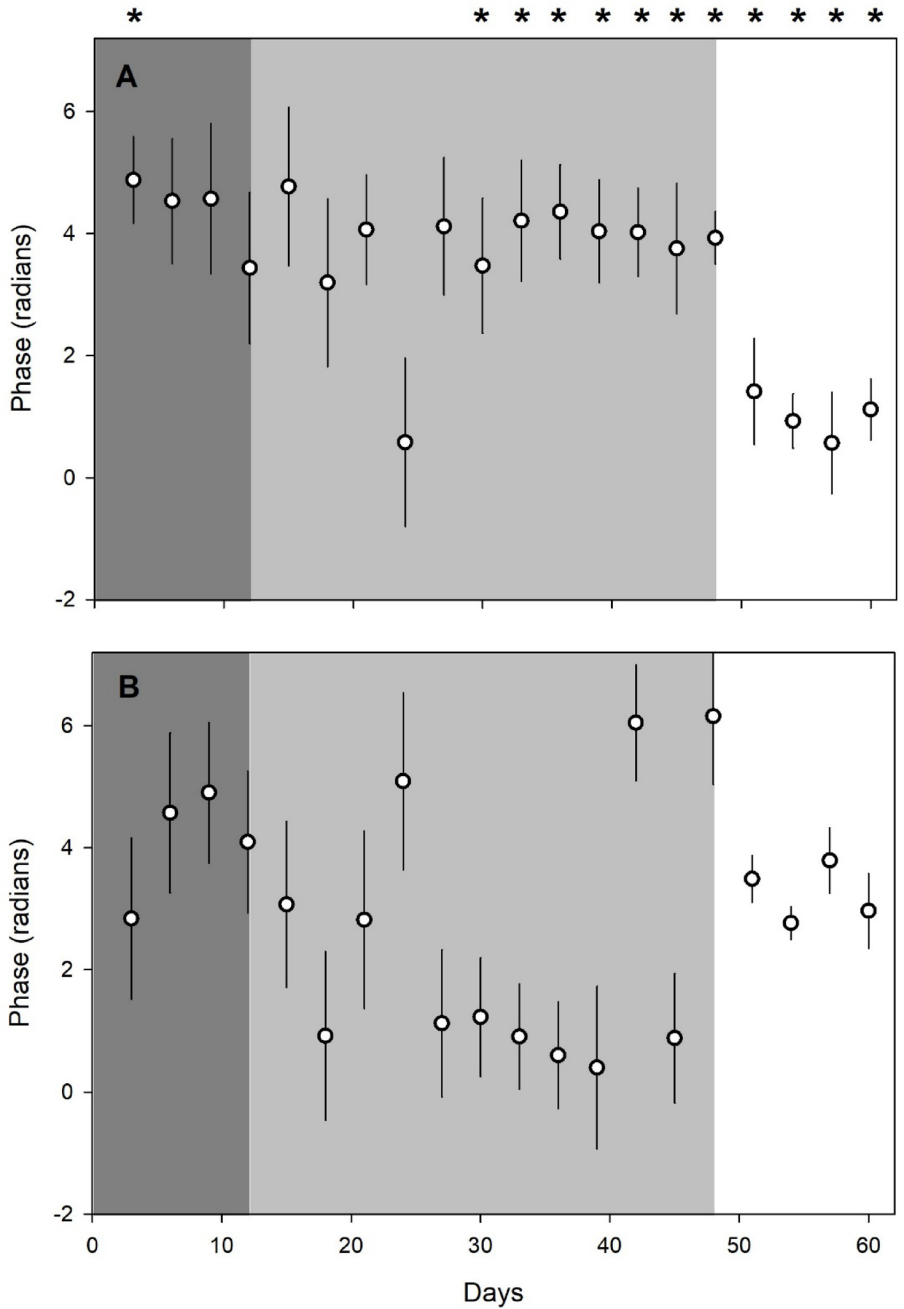
Median phases (± median deviation) for combined data from Winter 2023 and Winter 2024 experiments (see text for details), as determined from cosinor analysis with 3-d datasets. (**A**) Temperature phases; (**B**) CO_2_ concentration phases. Dark gray zone on left indicates complete darkness in CSU; light gray zone in center indicates period of 12 l:12D light regime; white zone on right indicates exterior (ambient) conditions outside CSU. Asterisks at the top of A) indicate statistically significant pairwise comparisons between hive temperature and CO_2_ concentration data.

**Fig. 7:**
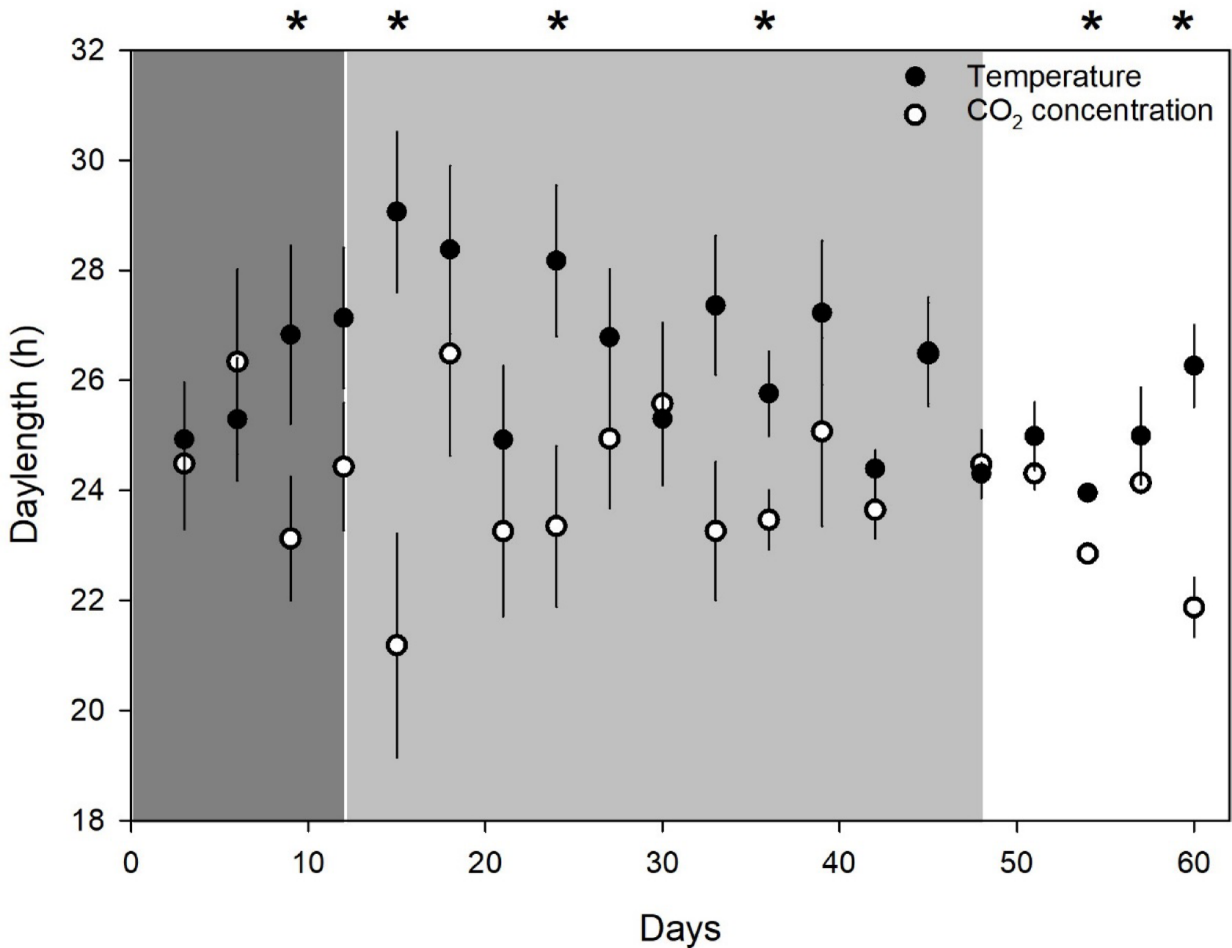
Average daylength as determined by cosinor analysis for combined data from Winter 2023 and Winter 2024 experiments (see text for details), as determined from cosinor analysis with 3-d datasets. (**A**) Temperature phases; (**B**) CO_2_ concentration phases. Dark gray zone on left indicates complete darkness in CSU; light gray zone in center indicates period of 12 l:12D light regime; white zone on right indicates exterior (ambient) conditions outside CSU. Asterisks at the top of A) indicate statistically significant pairwise comparisons between hive temperature and CO_2_ concentration data.

Phase and daylength data from the cosinor analysis (thus a value every 3 d) were combined across the Winter 2023 and Winter 2024 experiments to observe changes over time during periods in completely dark, 12 l:12D light regime, and outside conditions. After 15 d in the light regime, phases were significantly different between temperature and CO_2_ concentration, and they remained so, even after the CSU in outdoor conditions (Fig. 6, Supplementary Information Table S10). Period data differences were less consistent (Fig. 7).

## Discussion

Confirming circadian rhythms for any parameter involves (1) confirming the presence of the 24 h period of the target response variable both in the absence of environmental cues as well as across a range of temperatures, and (2) changing the phase of the observed rhythm. Honey bees are known to exhibit circadian rhythms in their individual locomotor activities, and phase shifts have been successfully introduced in circadian cycles of honey bee locomotor activity using an anaesthetic^4,5,21,22^. The focus of this study was on colony-level, rather than individual, activities. Colony-level activities were considered to be collective activities, such as foraging, brood rearing and thermoregulation, to achieve objectives that benefit the colony but do not necessarily apply to single bees. Foraging behavior and brood rearing would be less optimal response variables in this case because of their sensitivity to temperature: foragers will not fly when ambient temperatures are below about 12 °C, and brood rearing requires constant in-hive temperatures of about 34–35 °C (usually obtained only within the cluster). Furthermore, in order to manipulate environmental cues, such as light, for an entire colony the colony itself would need to be contained in an enclosed space. For colonies kept in enclosed spaces at temperatures above 12 °C, bees departing the hive would be attracted to any light source but unable to orientate their way back to the hive since they use the sun for navigation^23^. The resulting loss of a large proportion of adult workers would introduce confounding variables to the experiment.

Colonies can be kept safely for extended periods in enclosed spaces, such as a cold storage room, at temperatures below about 10 °C, but appropriate response variables for studies of circadian rhythms need to be selected. In these experiments we considered in-hive temperature and CO_2_ concentration to be proxy variables for the collective activities of thermoregulation and respiratory gas management within the hive. Both thermoregulation and CO_2_ management occur across a range of temperatures, and, by modifying the hive with an entrance cover to prevent losses of bees due to phototaxis, the lighting regime can be manipulated within the CSU to change rhythmic phases. While temperature and CO_2_ concentration are not themselves direct measures of activity, they are the product of a concerted colony activity. Measuring temperature and CO_2_ concentration would be considerably easier than measuring the activities of individual bees, and may shed light on the nature of collective behavior.

The questions addressed in this study were: (1) do temperature and CO_2_ concentration data cycles maintain approximate 24 h periods across a in conditions of constant temperature and darkness for extended periods; and (2) can the phases of those data be shown to be a function of external factors, and thus subject to change when those external factors change? The first question was addressed by comparing the periods of data from hives in cold temperatures (5 °C) for about 3 weeks with those of hives kept outside in ambient conditions (20–30 °C). Periodogram analyses, a common tool in circadian studies^24^, indicated that both temperature and CO_2_ concentration had strong, stable 24 h periods in both treatment groups. In the case of temperature, pairwise contrasts showed that the strength of the 24 h period declined over time in the CSU, but it remained the strongest signal in the detrended data. The strength of the 24 h period in temperature data did not change in a consistent fashion among hives kept outside. In the case of CO_2_ concentration, the strength of the 24 h period did not significantly change over time in the CSU, although some changes were observed among hives kept outside. These results show that the 24 h period was maintained across a range of temperatures for both parameters, and suggest that CO_2_ concentration may be a better parameter than temperature for studies on colony-level circadian rhythms.

The second question addressed here concerned the phases of the temperature and CO_2_ concentration data and whether those phases can be changed by altering external signals. Bee colonies were exposed to lighting cycles at a 12 h phase offset from ambient in an attempt to induce a phase shift. With respect to the Summer 2023 data, which included colonies kept in the CSU for 18 d as well as colonies kept outside at the same time, none of the phase comparisons between intervals of outside colonies was significant but most of the comparisons between intervals of CSU colonies were significant. This indicated that phases changed during the course of the experiment in the CSU but not outside. The variability of the phases, as measured by the magnitude of the standard deviations, was lower among colonies in the CSU than for those outside for temperature data, but higher among colonies in the CSU than those outside for CO_2_ concentration data. This same relationship, at least with respect to CO_2_ data, was observed in the Fall 2020 and Fall 2021 experiments. If light was a principle driver of the phase, and the quality and quantity of light was sufficient, then the light regime from 6PM to 6AM was expected to move the phases about π radians or about 12 h. The light regime caused the temperature phase to change by about 2.3 radians between interval 1 (under total darkness) and interval 3, about 2.1 radians in interval 3 between CSU colonies and outside colonies, and about 2.4 radians between interval 3 and the post-CSU interval (for consistency, all phase changes are expressed here as positive numbers). The light regime apparently had a reduced effect on CO_2_ concentration in this experiment, with differences of about 2.4 radians between intervals 1 and 3 in the CSU, about 1.4 radians in interval 3 between CSU colonies and outside colonies, and about 1.4 radians between interval 3 and post-CSU.

The durations of the Winter 2023 and Winter 2024 experiments were longer than that of the Summer 2023 experiments in order to determine whether more time in the lighting regime would have a stronger effect on temperature and CO_2_ concentration phases. The experiment was conducted in winter because that time of the year is ideal for maintaining bee colonies in cold storage facilities for long periods, and the duration of the initial constant dark period was 12 d, rather than 6 d for the Summer 2023 experiment. Three nonconsecutive intervals within the CSU were chosen for comparison, in addition to an interval post-CSU. No differences were observed with respect to the temperature phase among intervals within the CSU but all three were significantly different from the post-CSU interval, with phase changes ranging from 2.5 to 3.4 radians, or about 9 h 37 min to 12 h 58 min. With respect to the CO_2_ concentration contrasts, all pairwise contrasts were significant except one (interval 2 v. interval 3 within the CSU). The phase of interval 3 differed by about 4.1 radians (15 h 52 min) from that of interval 1, and about 3.1 radians (11 h 55 min) from that of the post-CSU interval. The rapid change in phase between interval 3 and the post-CSU interval in both the summer and winter experiments and with respect to both temperature and CO_2_ concentration may have been due to the abundance of environmental signals (temperature and humidity as well as light) for colonies in outside conditions compared to colonies in the CSU (light alone). The P values of the contrasts between interval 3 in the CSU and the post-CSU interval were < 0.001 in both Summer 2023 and the combined Winter 2023 and 2024 experiments, indicating a robust result. Considering only data from the cosinor analysis combined from the Winter 2023 and Winter 2024 experiments, phases did not differ significantly between temperature and CO_2_ concentration during the period of total darkness and during the initial part of the 12 l:12D light regime, suggesting that phases for both were highly variable. After that point, the colonies had presumably entrained to the light cycles and they were consistently significantly different. Average period length exhibited a different pattern of differences.

One aspect not completely addressed in this study is whether bee colonies can maintain circadian rhythms in the absence of external cues and across a range of constant temperatures. We examined a single constant temperature in this study. This aspect has two main issues: experimental design and data interpretation. Regarding experimental design, honey bee colonies can be safely kept for long periods in constant conditions for temperatures at or below 10 °C. Above that temperature, honey bees will try to leave the hive for foraging and other activities, and retaining them inside the hive for long periods will affect colony behavior and thus likely introduce confounding variables to the experiment. In addition, at temperatures below 0 °C the CSU will need to be designed to handle large amounts of water vapor produced by the hives, which was not the case here.

The second issue concerns exactly what is being measured in a bee hive in constant conditions of low temperature. Bee clusters in cold conditions are dynamic, with bees spending time in the insulation layer on the outside and more exposed to cold, before migrating inside the cluster and being replaced in the insulation layer by other bees^8^. In this sense a “superorganism” such as a bee colony is very different from, for example, a single organism. The components of the superorganism can freely move such that no component (bee) is actually exposed to a constant temperature for long periods, whereas the components (organs and tissues) of a single ectothermic organism are typically fixed relative to each other and after a period of time in a constant temperature environment would have the same temperature. Even if we consider only certain tissues or organs, such as neural tissue, in the bee colony, they are exposed to different temperatures in constant conditions at the same proportions as the bees themselves. Thus, the results presented here may not be materially different if the colonies were exposed to another constant low temperature, and that the bees that control temperature and CO_2_ concentration in the hive were unlikely to have done so while at the low, constant temperature themselves. The drive for honey bees to cluster to conserve temperature is strong; even caged bees in groups of as few as 50 will cluster at 15 °C and maintain temperatures above ambient^25^. Honey bee colonies reduce (but do not eliminate) thermoregulation effort in the absence of brood^19^ so a broodless colony at a constant temperature that it finds desirable may be the only instance of most or all bees having the same body temperature, but that is a very particular set of circumstances.

These results support the idea that colony-level behaviors such as thermoregulation and control of CO_2_ concentration are subject to circadian rhythms, although some aspects remain to be resolved, and those rhythms can be manipulated via external cues. Controlling temperature and respiration gases requires different kinds of worker activities, including resting in place (for example, as part of an insulating layer in a cluster) so how the associated rhythms manifest themselves on an individual scale is not clear. The rhythms in both cases appeared robust, even many days after an abrupt move into an environment of low temperature and total darkness. CO_2_ management appeared particularly stable under those conditions, confirming the view that regular occurrences of high CO_2_ concentrations are typical features of the hive environment.

## Materials and methods

All colonies were visually inspected for disease prior to being included in these experiments, and provided with sufficient food provisions.

### Fall 2020

In August 2020 forty bee colonies housed in two painted, 10-frame, wooden Langstroth boxes (43.7 l capacity) with marked European queens and at least 2 frames of sealed brood, were selected for the study^19^. Temperature sensors (iButton Thermochron, resolution ± 0.06 °C, accuracy ± 0.5 °C, accessed using 1-Wire Drivers x64, version 4.05) enclosed in plastic cassettes (Thermo Fisher Scientific, Waltham, MA) were stapled under the top bar on the middle frame in the bottom box and set to record every 30 min. Adult bee masses were measured on 16 September by subtracting the weight of all hive parts, including frames, lid, etc., from the total hive weight using a published protocol^26^. At the same time each side of all brood frames was photographed using a 16.3 megapixel digital camera (Canon Rebel SL1, Canon USA, Inc., Melville, NY). Frame photographs were analyzed to estimate the amount of sealed brood using ImageJ version 1.47 software (W. Rasband, National Institutes of Health, USA) or CombCount^27^ and frame values were summed to provide colony level estimates.

After the first assessment, hives were ranked with respect to adult bee mass and then assigned to a treatment group, with 20 hives per group, while ensuring that the mean colony bee masses were approximately equal between groups. CO_2_ probes (model GMP251, Vaisala Inc., Helsinki, Finland), calibrated for 0–20% concentrations and linked to HOBO UX120-006 M dataloggers set to record every 5 min, were placed on top of the center frames in the top box of 12 hives in each of the two groups. On 1 October migratory entrance screens were placed on one group of 20 colonies, which were then placed in a CSU (30 m^3^ internal volume, with CO_2_ and temperature monitors, PolarKing, Fort Wayne, IN) set to 5 °C with a dehumidifier and a roof-mounted exhaust fan operating 4 min per h. Red lights, undetectable by bees, were used during visits, and the door was covered with an opaque plastic curtain to eliminate any trace of light (the CSU was entered only at night). Ambient temperature and CO_2_ within the CSU were recorded every 5 min. Those hives remained in the unit with the entrance covers until 22 October. The remaining 20 hives (12 of which had CO_2_ sensors) were kept outside in the original apiary during that time. On 28 October hives were evaluated again.

### Fall 2021

 The experiment was repeated the following year, following largely the same schedule. Rainfall was considerably higher prior to the experiment, leading to abundant bee forage and to considerably larger colonies^19^. In August 2021, forty colonies were identified, placed on scales and treated with tau-fluvalinate (Apistan, Vita Bee Health, Basingstoke, UK). On 22 September all colonies were reduced to two boxes of 10 frames each; colonies contained on average more than twice as much brood as the previous year. On 27 September hive evaluations were conducted as in the previous year and hives were again divided into treatment groups as described for Fall 2020. On 4 October, migratory entrance screens were placed on all the hives in one treatment group and those hives were moved to the CSU, while half the hives remained outside in the original apiary. The ventilation fan was set to exchange air for 5 min per h to accommodate the larger bee mass compared to the previous year. Temperature was recorded every 15 min (rather than 30 min as in the previous year). The CSU was entered only on 5 October, to remove migratory entrance covers, and on 19 October for a data download. Hives were removed from the CSU after 3 weeks, as in the previous year. Hive were evaluated 26 October 2021.

### Summer 2023

 In May 2023 16 bee colonies with marked queens, housed in painted 10-frame Langstroth boxes and with at least 2 frames of sealed brood, were obtained. CO_2_ probes and temperature sensors (HOBO Pendant Temp MX2201, rather than iButtons) were installed as described above and set to record every 5 min. Colonies were evaluated as described above on 15 June. On 22 June eight of the colonies were moved into the CSU set to 5 °C. The ventilation fan was set to exchange air for 3 min per h, which was reduced from the previous experiments due to fewer colonies. Large mesh screens were placed over the entrance of each hive to provide space for the colony to dispose of dead bees but prevent bees from flying (Supplementary Information Figure S14). Four lights (BR40 LED, 1400 lumens, Sunco Lighting, Valencia CA) were installed on the ceiling. After 6 d darkness, light cycles turned on for 12 h from 6PM to 6AM each d for the remaining 12 d, after which those colonies were moved out of the CSU and back to their original apiary. On 11 July the brood frame photographs were taken but the adult bee mass was estimated visually as frames of bees (hereafter “assessment”)^28^. On 14 July the remaining eight colonies were moved into the CSU and subjected to the same treatment: 6 d darkness followed by 12 d of 12:12 light: dark cycle, with light from 6PM to 6AM. Those colonies were removed from the CSU to the outdoor apiary on 2 August and assessed on 3 August as described above for the first group of colonies. To estimate the adult bee mass for the post-CSU assessments, adult bee mass in kg was regressed on frames of bees using data from the pre-CSU evaluation, and the resulting equation used to convert frames of bees to adult bee mass.

### Winter 2023

 On 7 December 2023 eight bee colonies with marked queens were assessed by taking photographs of brood frames, as described for Summer 2023, with a visual count of frames of bees (a full assessment was contra-indicated because of cold weather). All hives were equipped with temperature and CO_2_ sensors as described for Summer 2023. On 8 December the large mesh screens were placed over hive entrances and the hives placed in the CSU at 5 °C as described above. Colonies were initially subjected to 12 d of complete darkness. On 20 December the same daily lighting schedule used for the experiments in Summer 2023 was initiated, i.e. 12 h light from 6PM to 6AM, followed by 12 h dark. All colonies were removed on 25 January 2024, placed in their original locations in the apiary, and evaluated on 26 January. Frames of bee data from the assessment on 7 December were used to estimate adult bee mass in kg prior to the CSU treatment using the method described above.

### Winter 2024

 The Winter 2023 experiment was replicated. On 4 December 2024 ten bee colonies with marked queens were assessed by taking photographs of brood frames with a visual count of frames of bees, and hives were equipped with temperature and CO_2_ sensors as described for Winter 2023. On 6 December hives were placed in the CSU at 5 °C with screened hive entrances as described above. Colonies were again subjected to 12 d of complete darkness. On 21 December the same daily lighting schedule used for the Winter 2023 experiment was initiated. All colonies were removed on 27 January 2025, placed in their original locations in the apiary, and evaluated on 28 January. As before, frames of bee data from the assessment on 4 December were used to estimate adult bee mass in kg prior to the CSU treatment.

### Data analysis

 Raw temperature and CO_2_ concentration data for all datasets were detrended by subtracting the 48 h running average from the raw data. The 48 h running average was chosen to remove most of the information associated with periods greater than 48 h. The detrended data were then divided into six 3-d subsets. 3-d subsets were considered sufficiently long to characterize colony behavior yet short enough to maximize the number of data points. For the Fall 2020 and Fall 2021 datasets, each 3-d subset was subjected to a periodogram analysis using the periodogram function in the TSA package^29,30^ in R. Periodograms are expressed in the frequency domain rather than the time domain. A periodogram analysis decomposes a signal, in this case temperature and CO_2_ concentration, into discrete frequencies of sinusoidal components and provides an estimate of the contribution of each frequency to the signal in the form of the power spectral density^29^. The power spectral density divided by the product of the variance and (N-1), where N is the number of observations in a sample, produces the proportion sums of squares contributed by each frequency. The total sums of squares contributions is equal to one for the analysis of each sample, so the relative importance of each frequency is easy to judge. To examine whether the strength of the 24 h period changed over time or differed between experiments, data were first examined for normality. As the data were not normally distributed and normality could not be achieved through data transformation, data were pooled across the two experiments and pairwise nonparametric two-tailed Wilcoxon signed rank tests were conducted (wilcox.test function in R). Contrasts were grouped into four families: CO_2_ and temperature data within the CSU and outside the CSU (ambient), and a Bonferroni correction applied to each contrast family. To better estimate the daily period during darkness and constant temperature, data from the first 6 d of the Summer 2023 experiments, and the second 6 d of the Winter 2023 and Winter 2024 experiments (when temperature and CO_2_ were measured every 5 min) were subjected to a Lomb-Scargle periodogram analysis (ClockLab 6.1.15, Actimetrics Software) and restricted to periods < 30 h with the highest amplitudes. Lomb-Scargle periodograms have been found superior to chi-square periodograms in many instances^31^.

Cosine curves were fit to the detrended data using R cosinor package^32^, with acrophases corrected and curve fitness tested, using functions from the R cosinor2 package^33^. Data from Summer 2023, Winter 2023 and Winter 2024 experiments were examined for phase changes due to the lighting schedule. Data for the Summer 2023 experiment were treated as follows: the 18 d of data from within the CSU and 6 d of post-CSU data were divided into 8 consecutive subsets of 3 d each, with each subset subjected to a cosinor analysis. The 8 subsets were then considered as four “intervals” of 2 subsets each: interval 1 consisted of days 1–6 (dark in CSU); interval 2 consisted of days 7–12 (12 h light phase in CSU); interval 3 consisted of days 13–18 (12 h light phase in CSU); and the post-CSU interval consisted of days 19–24 (post-CSU in outdoor apiary). Thus each interval contained the pooled data of 16 colonies across two subsets, or 32 data points. A Watson’s goodness of fit test for the von Mises distribution, considered the normal distribution for circular statistics, was conducted on each interval of data using the circular package in R^34^. Watson’s test for homogeneity on two samples was performed on each pairwise comparison among the four intervals using the same R package; this nonparametric test performed well compared to other common tests of circular statistics^35^. Data were pooled because combined sample sizes in excess of 17 are recommended for the Watson’s test for homogeneity^34^.

Data from the Winter 2023 and Winter 2024 experiments involved colonies kept in the CSU for 45 d and those data were analyzed in similar fashion. Data were again divided into four intervals, with each interval again consisting of two, 3-d subsets: interval 1 consisted of days 7–12 (dark in CSU); interval 2 consisted of days 25–30 (12 h light regime in CSU); interval 3 consisted of days 40–45 (12 h light regime in CSU); and the post-CSU interval consisted of days 52–57 (post-CSU in outdoor apiary). As with the Summer 2023 data, Watson tests for goodness of fit and for homogeneity on two samples were conducted on all intervals. Rose diagrams of the phases of each interval were produced using the circular package^34^. Changes in phase and period for the combined Winter 2023 and Winter 2024 data were compared between hive temperature and CO_2_ concentration over time wtih pairwise comparisons within 3-d samples. Watson 2-sample tests were used for phase data and Kruskal-Wallis tests were used for period data.

## Electronic supplementary material

Below is the link to the electronic supplementary material.

Supplementary Material 1.

## Data Availability

The raw temperature and CO2 data, and hive evaluation data, generated in this study can be found at: Meikle, William (2024) Circadian rhythm studies - temperature, CO2 and colony size data. Ag Data Commons. Dataset. https://doi.org/10.15482/USDA.ADC/26835739. All statistical results are available in the Supplementary Information.
