# Supplementary material for: Temperature and CO2 concentration in honey bee hives exhibit circadian rhythms
Source: Sci Rep. 2025 Jul 1;15:22042. doi: 10.1038/s41598-025-03614-3 (PMC12219277; doi:10.1038/s41598-025-03614-3)
Supplement: Supplementary file 1 — Supplementary Material 1. [file 41598_2025_3614_MOESM1_ESM.docx]

**Supplemental material**

**Colony-level controls of temperature and CO_2_ concentration in honey bee hives exhibit circadian rhythms**

W.G. Meikle and M. Weiss

**Table S1**. Activity log for cold storage unit (CSU) experiments.

| **Experiment** | **Date** | **Activity** |
| --- | --- | --- |
| Fall 2020 | 16 September | Hives evaluated |
|  | 1 October | Hives moved into CSU |
|  | 4 October | CSU entered for inspection |
|  | 8 October | CSU entered for data download |
|  | 22 October | Hives moved from CSU back to outside apiary |
|  | 28 October | Hives evaluated |
|  |  |  |
| Fall 2021 | 27 September | Hives evaluated |
|  | 4 October | Hives moved into CSU |
|  | 5 October | CSU hives checked |
|  | 19 October | CSU entered for data download |
|  | 24 October | Hives moved from CSU back to outside apiary |
|  | 26 October | Hives evaluated |
|  |  |  |
| Summer 2023 | 15 June | All hives evaluated |
|  | 22 June | Screened entrances installed, 8 hives moved into CSU |
|  | 28 June | CSU entered to turn on light timer |
|  | 29 June | CSU entered at 6:30PM for inspection |
|  | 1 July | CSU entered during light phase to clear dead bees. |
|  | 10 July | Hives moved from CSU back to outside apiary |
|  | 11 July | CSU hives assessed (see text for details) |
|  | 14 July | Screened entrances installed, remaining 8 hives moved into CSU |
|  | 20 July | CSU entered to turn on light timer |
|  | 2 August | Hives moved from CSU back to outside apiary |
|  | 3 August | CSU hives assessed |
|  |  |  |
| Winter 2023 | 7 December | All hives assessed |
|  | 8 December | Screened entrances installed, 8 hives moved into CSU |
|  | 20 December | CSU entered to turn on light timer and inspect hives |
|  | 22 December | CSU entered during light phase to check timer |
|  | 25 January | Hives moved from CSU back to outside apiary |
|  | 26 January | Hives evaluated |

**Table S2**. Average adult bee masses and brood surface areas for bee colonies before and after cold storage treatment.

| Experiment | Location | Before | | |  | After | | |
| --- | --- | --- | --- | --- | --- | --- | --- | --- |
|  |  | N | Adult mass (kg) | Brood area (cm^2^) |  | N | Adult mass (kg) | Brood area (cm^2^) |
| Fall 2020 | CSU | 21 | 1.41±0.11 | 916±106 |  | 21 | 1.00±0.09 | 40±10 |
|  | Outside | 24 | 1.35±0.09 | 1002±72 |  | 24 | 1.07±0.08 | 559±23 |
|  |  |  |  |  |  |  |  |  |
| Fall 2021 | CSU | 20 | 3.89±0.19 | 2453±122 |  | 20 | 4.24±0.25 | 7±3 |
|  | Outside | 18 | 4.03±0.20 | 2362±191 |  | 18 | 2.57±0.15 | 695±126 |
|  |  |  |  |  |  |  |  |  |
| Summer 2023 | CSU | 16 | 2.88±0.26 | 2223±265 |  | 16 | 2.42±0.13^a^ | 220±54 |
|  |  |  |  |  |  |  |  |  |
| Winter 2023 | CSU | 8 | 2.07±0.15^a^ | 527±68 |  | 8 | 1.44±0.14 | 0 |
|  |  |  |  |  |  |  |  |  |
| Winter 2024 | CSU | 10 | 1.54±0.13^a^ | 788±228 |  | 6-8 | 0.86±0.22 | 0 |

^a^ Estimated from visual inspection (see Materials and Methods for details).

**Table S3.** Results of pairwise Wilcoxon contrasts (Group 1 – Group 2) of the strength of the 24-hour signal for temperature and CO_2_ concentration data within the CSU and outside the CSU (ambient) for experiments conducted in 2020 and 2021. N=45 pairs for each contrast. “Contrast” indicates which of the 3-d subsets were compared, with subsets from 1-6; “Stat” is the Wilcoxon test statistic; and “Prob” is the statistical probability. With α=0.05 for each of the four families of contrasts, α_contrast_= 0.05/15=0.0033. Significant comparisons are shown in bold.

| **Pairwise contrast** | |  | **Temperature CSU** | |  | **Temperature out** | |  | **CO_2_ CSU** | |  | **CO_2_ out** | |
| --- | --- | --- | --- | --- | --- | --- | --- | --- | --- | --- | --- | --- | --- |
| **Subset 1** | **Subset 2** |  | **Stat** | **Prob** |  | **Stat** | **Prob** |  | **Stat** | **Prob** |  | **Stat** | **Prob** |
| 1 | 2 |  | **841** | **< 0.001** |  | 422 | 0.546 |  | 127 | 0.527 |  | 139 | 0.988 |
| 1 | 3 |  | **1007** | **< 0.001** |  | 369 | 0.214 |  | 144 | 0.877 |  | **273** | **< 0.001** |
| 1 | 4 |  | **1031** | **< 0.001** |  | 388 | 0.311 |  | 124 | 0.473 |  | 155 | 0.622 |
| 1 | 5 |  | **1035** | **< 0.001** |  | 257 | 0.008 |  | 125 | 0.491 |  | 161 | 0.501 |
| 1 | 6 |  | **891** | **< 0.001** |  | 286 | 0.023 |  | 88 | 0.079 |  | **251** | **< 0.001** |
| 2 | 3 |  | **1006** | **< 0.001** |  | 377 | 0.252 |  | 118 | 0.375 |  | **267** | **< 0.001** |
| 2 | 4 |  | **1017** | **< 0.001** |  | 354 | 0.154 |  | 140 | 0.790 |  | 170 | 0.345 |
| 2 | 5 |  | **1024** | **< 0.001** |  | **229** | **0.003** |  | 121 | 0.422 |  | 172 | 0.315 |
| 2 | 6 |  | **857** | **< 0.001** |  | **229** | **0.003** |  | 95 | 0.121 |  | **242** | **0.001** |
| 3 | 4 |  | **792** | **0.002** |  | 425 | 0.570 |  | 123 | 0.456 |  | **2** | **< 0.001** |
| 3 | 5 |  | **788** | **0.002** |  | 287 | 0.024 |  | 122 | 0.439 |  | **7** | **< 0.001** |
| 3 | 6 |  | 614 | 0.042 |  | 292 | 0.028 |  | 92 | 0.101 |  | 99 | 0.247 |
| 4 | 5 |  | 613 | 0.287 |  | **227** | **0.002** |  | 126 | 0.509 |  | 148 | 0.777 |
| 4 | 6 |  | 469 | 0.833 |  | 240 | 0.004 |  | 97 | 0.136 |  | **262** | **< 0.001** |
| 5 | 6 |  | 354 | 0.228 |  | 465 | 0.929 |  | 136 | 0.705 |  | **255** | **< 0.001** |

**Table S4.** Descriptive statistics for the phase values for continuous temperature and CO_2_ data for the Fall 2020 and Fall 2021 experiments. “Interval” indicates the 6 d interval after the start of the experiment, with each interval consisting of two 3-d subsets. “No. colonies” indicates the number of colonies in that treatment group across the two years of the study. “Colony site” indicates whether the data are from colonies within the CSU (“CSU”) or outside in ambient conditions (“out”). “Fit statistic” refers to a Watson’s goodness of fit test for the von Mises distribution. Distributions significantly different from a von Mises distribution are in bold. N=32 for each interval.

| **Parameter** | **Colony site** | **Interval** | **No. colonies** | **Mean ±**  **stand. dev.** | **Median** | **Fit statistic** | **P** |
| --- | --- | --- | --- | --- | --- | --- | --- |
| Temperature | CSU | 1-6 d | 90 | 5.08±0.51 | 5.13 | 0.07 | > 0.10 |
|  | CSU | 7-12 d | 90 | 5.01±1.09 | 5.01 | 0.04 | > 0.10 |
|  | CSU | 13-18 d | 90 | 5.33±1.98 | 5.32 | 0.03 | > 0.10 |
|  | out | 1-6 d | 86 | 2.35±2.04 | 2.11 | **0.45** | **< 0.01** |
|  | out | 7-12 d | 86 | 2.40±1.67 | 2.09 | **0.24** | **< 0.01** |
|  | out | 13-18 d | 86 | 1.38±1.61 | 1.51 | **0.34** | **< 0.01** |
|  |  |  |  |  |  |  |  |
| CO_2_ | CSU | 1-6 d | 48 | 4.63±1.88 | 4.69 | **0.17** | **< 0.01** |
|  | CSU | 7-12 d | 48 | 0.36±2.22 | 0.34 | 0.03 | > 0.10 |
|  | CSU | 13-18 d | 48 | 4.50±1.37 | 4.43 | 0.05 | > 0.10 |
|  | out | 1-6 d | 46 | 5.66±0.49 | 5.65 | 0.10 | > 0.05 |
|  | out | 7-12 d | 46 | 5.59±0.74 | 5.71 | 0.06 | > 0.10 |
|  | out | 13-18 d | 46 | 5.73±0.65 | 5.76 | 0.05 | > 0.10 |

**Table S5**. Descriptive statistics for the phase values for continuous temperature and CO_2_ data for the Summer 2023 experiment involving 16 honey bee colonies. “Interval” indicates the 6 d interval, with each interval consisting of two 3-d subsets. “Colony site” indicates whether the data are from colonies within the CSU (“CSU”) or outside in ambient conditions (“out”). “Post” indicates two 3-d subsets of data collected from colonies just after they had been moved out of the CSU. “Fit statistic” refers to a Watson’s goodness of fit test for the von Mises distribution. Distributions significantly different from a von Mises distribution are in bold. N=32 for each interval.

| **Parameter** | **Colony site** | **Interval** | **Mean ± stand. dev.** | **Median** | **Fit statistic** | **P** |
| --- | --- | --- | --- | --- | --- | --- |
| Temperature | CSU | 1-6 d | 4.40±0.32 | 4.41 | 0.028 | > 0.10 |
|  | CSU | 7-12 d | 3.69±0.86 | 3.99 | **0.247** | **< 0.01** |
|  | CSU | 13-18 d | 2.12±0.60 | 2.10 | 0.053 | > 0.10 |
|  | out | post | 4.54±1.22 | 4.79 | **0.163** | **< 0.01** |
|  | out | 1-6 d | 3.77±1.38 | 3.83 | 0.055 | > 0.10 |
|  | out | 7-12 d | 4.19±1.46 | 4.25 | 0.055 | > 0.10 |
|  | out | 13-18 d | 4.18±1.52 | 4.44 | 0.062 | > 0.05 |
|  |  |  |  |  |  |  |
| CO_2_ | CSU | 1-6 d | 5.67±1.61 | 5.66 | 0.028 | > 0.10 |
|  | CSU | 7-12 d | 3.34±1.35 | 3.40 | **0.083** | **< 0.05** |
|  | CSU | 13-18 d | 3.46±1.57 | 3.53 | 0.050 | > 0.10 |
|  | out | post | 4.74±0.36 | 4.73 | **0.153** | **< 0.05** |
|  | out | 1-6 d | 4.91±0.60 | 4.91 | **0.352** | **< 0.01** |
|  | out | 7-12 d | 5.04±0.45 | 5.00 | 0.046 | > 0.10 |
|  | out | 13-18 d | 4.86±0.35 | 4.86 | **0.142** | **< 0.05** |

**Table S6**. Descriptive statistics for the phase values for continuous temperature and CO_2_ data for the Winter 2023 experiment. “Interval” indicates the 6-d interval, with each interval consisting of two 3-d subsets. “Colony site” indicates whether the data are from colonies within the CSU (“CSU”) or outside in ambient conditions (“out”). “Post” indicates two 3-d subsets of data collected from colonies just after they had been moved out of the CSU. “Fit statistic” refers to a Watson’s goodness of fit test for the von Mises distribution. Distribution significantly different from a von Mises distribution is in bold. N=32 for each interval.

| **Parameter** | **Colony site** | **Interval** | **Mean ± stand. dev.** | **Median** | **Fit statistic** | **P** |
| --- | --- | --- | --- | --- | --- | --- |
| Temperature | CSU | 1-6 d | 4.21±1.77 | 4.00 | 0.065 | > 0.05 |
|  | CSU | 7-12 d | 3.94±1.64 | 4.06 | 0.025 | > 0.10 |
|  | CSU | 13-18 d | 4.50±1.25 | 4.36 | 0.038 | > 0.10 |
|  | out | post | 0.63±1.12 | 0.68 | **0.112** | **< 0.05** |
|  |  |  |  |  |  |  |
| CO_2_ | CSU | 1-6 d | 4.89±1.46 | 4.90 | 0.027 | > 0.10 |
|  | CSU | 7-12 d | 3.10±1.70 | 2.87 | 0.030 | > 0.10 |
|  | CSU | 13-18 d | 0.79±1.34 | 0.88 | 0.046 | > 0.10 |
|  | out | post | 3.32±0.79 | 3.25 | 0.062 | > 0.10 |

**Table S7.** Contrast values (Group 1 – Group 2) for comparisons of phases among periods within sites and sites within period for the Fall 2020 and Fall 2021 experiments. “Interval” indicates the 6-d interval, with each interval consisting of two 3-d subsets. “Contrast family” indicates the groups being compared, both across intervals within site, and within intervals across site, with site being within the CSU (“CSU”) or outside in ambient conditions (“Outside”). “Watson” indicates the value of the Watson’s test for homogeneity on two samples of circular data, “N1” and “N2” refer to the number of colonies in each group of the contrast, and “P” refers to the probability of the statistic value. Significant contrasts are in bold.

| **Parameter** | **Contrast family** | **Interval 1** | **Interval 2** | **Watson** | **N1** | **N2** | **P** |
| --- | --- | --- | --- | --- | --- | --- | --- |
| Temperature | CSU | 1-6 d | 7-12 d | **0.749** | **90** | **90** | **< 0.001** |
|  |  | 1-6 d | 13-18 d | **1.760** | **90** | **90** | **< 0.001** |
|  |  | 7-12 d | 13-18 d | **0.487** | **90** | **90** | **< 0.001** |
|  | Outside | 1-6 d | 7-12 d | 0.050 | 86 | 86 | > 0.05 |
|  |  | 1-6 d | 13-18 d | **0.338** | **86** | **86** | **< 0.01** |
|  |  | 7-12 d | 13-18 d | **0.288** | **86** | **86** | **< 0.01** |
|  | CSU vs Outside | 1-6 d | 1-6 d | **1.380** | **90** | **86** | **< 0.001** |
|  |  | 7-12 d | 7-12 d | **1.049** | **90** | **86** | **< 0.001** |
|  |  | 13-18 d | 13-18 d | **0.392** | **90** | **86** | **< 0.001** |
|  |  |  |  |  |  |  |  |
| CO_2_ | CSU | 1-6 d | 7-12 d | 0.181 | 48 | 48 | > 0.05 |
|  |  | 1-6 d | 13-18 d | 0.192 | 48 | 48 | < 0.05 |
|  |  | 7-12 d | 13-18 d | **0.278** | **48** | **48** | **< 0.01** |
|  | Outside | 1-6 d | 7-12 d | 0.126 | 46 | 46 | > 0.05 |
|  |  | 1-6 d | 13-18 d | 0.054 | 46 | 46 | > 0.05 |
|  |  | 7-12 d | 13-18 d | 0.050 | 46 | 46 | > 0.05 |
|  | CSU vs Outside | 1-6 d | 1-6 d | **1.165** | **48** | **46** | **< 0.001** |
|  |  | 7-12 d | 7-12 d | **0.765** | **48** | **46** | **< 0.001** |
|  |  | 13-18 d | 13-18 d | **0.638** | **48** | **46** | **< 0.001** |

**Table S8.** Contrast values (Group 1 – Group 2) for comparisons of phases among periods within sites and sites within period for Summer 2023. “Interval” indicates the 6-d interval, with each interval consisting of two 3-d subsets. “Contrast family” indicates the groups being compared, both across intervals within site, and within intervals across site, with site being within the CSU (“CSU”) or outside in ambient conditions (“Outside”). “Post” indicates two 3-d subsets of data collected from colonies just after they had been moved out of the CSU. “Watson” indicates the value of the Watson’s test for homogeneity on two samples of circular data, and “P” refers to the probability of the value. N=32 for each interval of each contrast. Significant contrasts are in bold.

| **Parameter** | **Contrast family** | **Interval 1** | **Interval 2** | **Contrast value** | **Watson** | **P** |
| --- | --- | --- | --- | --- | --- | --- |
| Temperature | CSU | 1-6 d | 7-12 d | **0.703** | **0.248** | **< 0.05** |
|  |  | 1-6 d | 13-18 d | **2.279** | **1.222** | **< 0.001** |
|  |  | 1-6 d | post | **-0.142** | **0.752** | **< 0.001** |
|  |  | 7-12 d | 13-18 d | **1.576** | **0.833** | **< 0.001** |
|  |  | 7-12 d | post | **-0.845** | **0.455** | **< 0.001** |
|  |  | 13-18 d | post | **-2.421** | **0.888** | **< 0.001** |
|  | Outside | 1-6 d | 7-12 d | -0.419 | 0.060 | > 0.10 |
|  |  | 1-6 d | 13-18 d | -0.411 | 0.053 | > 0.10 |
|  |  | 7-12 d | 13-18 d | 0.008 | 0.052 | > 0.10 |
|  | CSU vs Outside | 1-6 d | 1-6 d | **0.625** | **0.480** | **< 0.001** |
|  |  | 7-12 d | 7-12 d | **-0.497** | **0.252** | **< 0.05** |
|  |  | 13-18 d | 13-18 d | **-2.065** | **0.783** | **< 0.001** |
|  |  |  |  |  |  |  |
| CO_2_ | CSU | 1-6 d | 7-12 d | **2.330** | **0.394** | **< 0.001** |
|  |  | 1-6 d | 13-18 d | **2.214** | **0.221** | **< 0.05** |
|  |  | 1-6 d | post | **0.938** | **0.985** | **< 0.001** |
|  |  | 7-12 d | 13-18 d | -0.116 | 0.059 | > 0.10 |
|  |  | 7-12 d | post | **-1.392** | **1.048** | **< 0.001** |
|  |  | 13-18 d | post | **-1.276** | **0.993** | **< 0.001** |
|  | Outside | 1-6 d | 7-12 d | -0.128 | 0.110 | > 0.10 |
|  |  | 1-6 d | 13-18 d | 0.044 | 0.115 | > 0.10 |
|  |  | 7-12 d | 13-18 d | 0.172 | 0.062 | > 0.10 |
|  | CSU vs Outside | 1-6 d | 1-6 d | **0.766** | **0.747** | **< 0.001** |
|  |  | 7-12 d | 7-12 d | **-1.692** | **1.187** | **< 0.001** |
|  |  | 13-18 d | 13-18 d | **-1.404** | **1.008** | **< 0.001** |

**Table S9.** Contrast values (Group 1 – Group 2) for comparisons of phases among periods within sites and sites within period for both winter experiments (2023-24 and 2024-25). “Interval” indicates the 12-d interval, with each interval consisting of four 3-d subsets. “Site” indicates whether the data are from colonies within the CSU (“CSU”) or outside in ambient conditions (“out”) and “post” indicates four 3-d subsets of data collected from colonies after they had been moved out of the CSU. “Watson” indicates the value of the Watson’s test for homogeneity on two samples of circular data, and “P” refers to the probability of the value. N=32 for each interval of each contrast. Significant contrasts are in bold.

| **Parameter** | **Group 1** | |  | **Group 2** | | **Contrast value** | **Watson** | **P** |
| --- | --- | --- | --- | --- | --- | --- | --- | --- |
|  | **Site** | **Interval** |  | **Site** | **Interval** |  |  |  |
| Temperature | CSU | 1-12 d |  | CSU | 19-30 d | 6.079 | 0.069 | >0.1 |
|  | CSU | 1-12 d |  | CSU | 34-45 d | 0.835 | 0.146 | >0.1 |
|  | CSU | 1-12 d |  | out | post | **3.213** | **1.084** | **<0.001** |
|  | CSU | 19-30 d |  | CSU | 34-45 d | 1.039 | 0.173 | >0.05 |
|  | CSU | 19-30 d |  | out | post | **3.417** | **1.058** | **<0.001** |
|  | CSU | 34-45 d |  | out | post | **2.379** | **1.634** | **<0.001** |
|  |  |  |  |  |  |  |  |  |
| CO_2_ | CSU | 1-12 d |  | CSU | 19-30 d | **3.256** | **0.290** | **<0.01** |
|  | CSU | 1-12 d |  | CSU | 34-45 d | **3.954** | **0.537** | **<0.001** |
|  | CSU | 1-12 d |  | out | post | **3.137** | **0.938** | **<0.001** |
|  | CSU | 19-30 d |  | CSU | 34-45 d | 0.697 | 0.146 | >0.1 |
|  | CSU | 19-30 d |  | out | post | **3.137** | **1.013** | **<0.001** |
|  | CSU | 34-45 d |  | out | post | **2.440** | **1.475** | **<0.001** |

**Table S10.** Watson 2-sample comparisons of phases and Kruskal-Wallis comparisons of periods, as calculated using a cosinor analysis, between hive temperature and CO_2_ concentration data from the combined Winter 2023 and Winter 2024 data. “Days” refers to the 3-d interval after bee hives were placed in the CSU, “Watson statistic” refers to the result of the Watson 2-sample test, and “Temp. d.f.” and “CO_2_ d.f.” refer to the degrees of freedom associated with the temperature and CO_2_ concentration data, respectively. Significant contrasts (P,0.05) are in bold.

| Days | Temp. d.f. | CO_2_ d.f. | Phase data | |  | Period data | |
| --- | --- | --- | --- | --- | --- | --- | --- |
|  |  |  | Watson statistic | Prob. |  | Chi-square statistic | Prob. |
| 1-3 | 18 | 18 | **0.347** | **<0.01** |  | 0.0491 | 0.8246 |
| 4-6 | 18 | 18 | 0.045 | >0.1 |  | 0.2729 | 0.6014 |
| 7-9 | 18 | 18 | 0.025 | >0.1 |  | **4.2309** | **0.0397** |
| 10-12 | 18 | 18 | 0.092 | >0.1 |  | 1.3011 | 0.254 |
| 13-15 | 18 | 18 | 0.113 | >0.1 |  | **6.3733** | **0.0116** |
| 16-18 | 18 | 18 | 0.061 | >0.1 |  | 0.4898 | 0.484 |
| 19-21 | 18 | 18 | 0.116 | >0.1 |  | 0.5787 | 0.4468 |
| 22-24 | 18 | 18 | 0.033 | >0.1 |  | **4.643** | **0.0312** |
| 25-27 | 18 | 18 | 0.158 | >0.05 |  | 0.4214 | 0.5162 |
| 28-30 | 18 | 18 | **0.229** | **<0.05** |  | 0.4427 | 0.5058 |
| 31-33 | 18 | 18 | **0.404** | **<0.001** |  | 3.037 | 0.0814 |
| 34-36 | 18 | 18 | **0.469** | **<0.001** |  | **4.233** | **0.0396** |
| 37-39 | 18 | 18 | **0.207** | **<0.05** |  | 1.138 | 0.2861 |
| 40-42 | 18 | 18 | **0.387** | **<0.001** |  | 1.7266 | 0.1888 |
| 43-45 | 18 | 18 | **0.249** | **<0.05** |  | 0.0424 | 0.8369 |
| 46-48 | 10 | 10 | **0.283** | **<0.01** |  | 0.6314 | 0.4268 |
| 49-51 | 15 | 13 | **0.529** | **<0.001** |  | 0.0085 | 0.9265 |
| 52-54 | 15 | 13 | **0.586** | **<0.001** |  | **16.7393** | **<0.0001** |
| 55-57 | 15 | 13 | **0.459** | **<0.001** |  | 3.1714 | 0.0749 |
| 58-60 | 15 | 13 | **0.556** | **<0.001** |  | **10.4207** | **0.0012** |


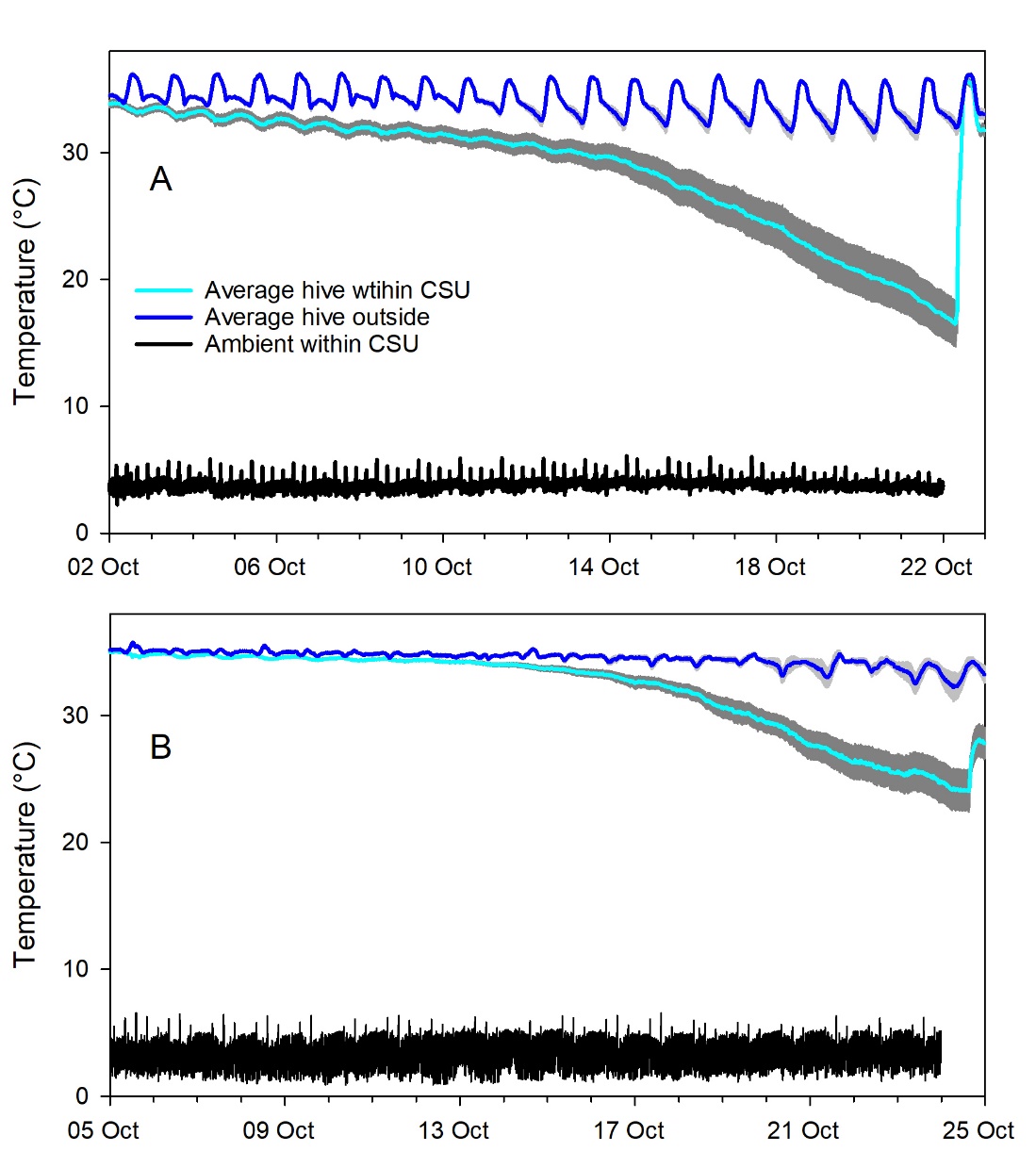


**Figure S1.** Raw temperature data for colonies inside the CSU and outside. A) Fall 2020 experiment; B) Fall 2021 experiment. Standard errors are shown in gray. Hives in the 2020 experiment had migratory entrance screens but those were removed on 5 October in the 2021 experiment. A fan evacuated air for 4 minutes per h in the 2020 experiment and 5 minutes per h in the 2021 experiment.


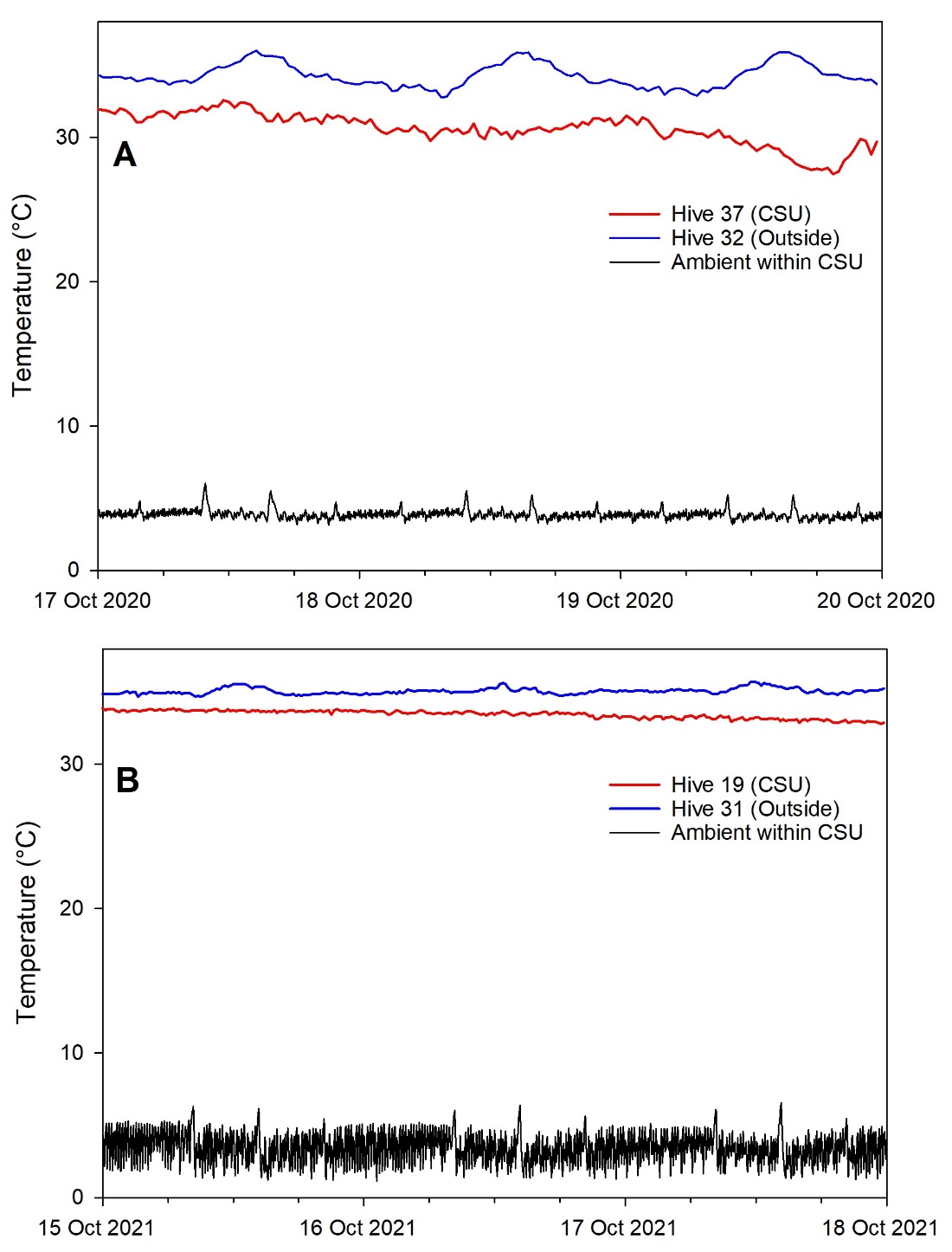


**Figure S2.** Sample temperature data over 3 d after 2 weeks of storage. A) Fall 2020 experiment (hive data collected every 30 min. and ambient every 5 min. – see text for details); B) Fall 2021 experiment (hive data collected every 15 min. and ambient every 5 min.).


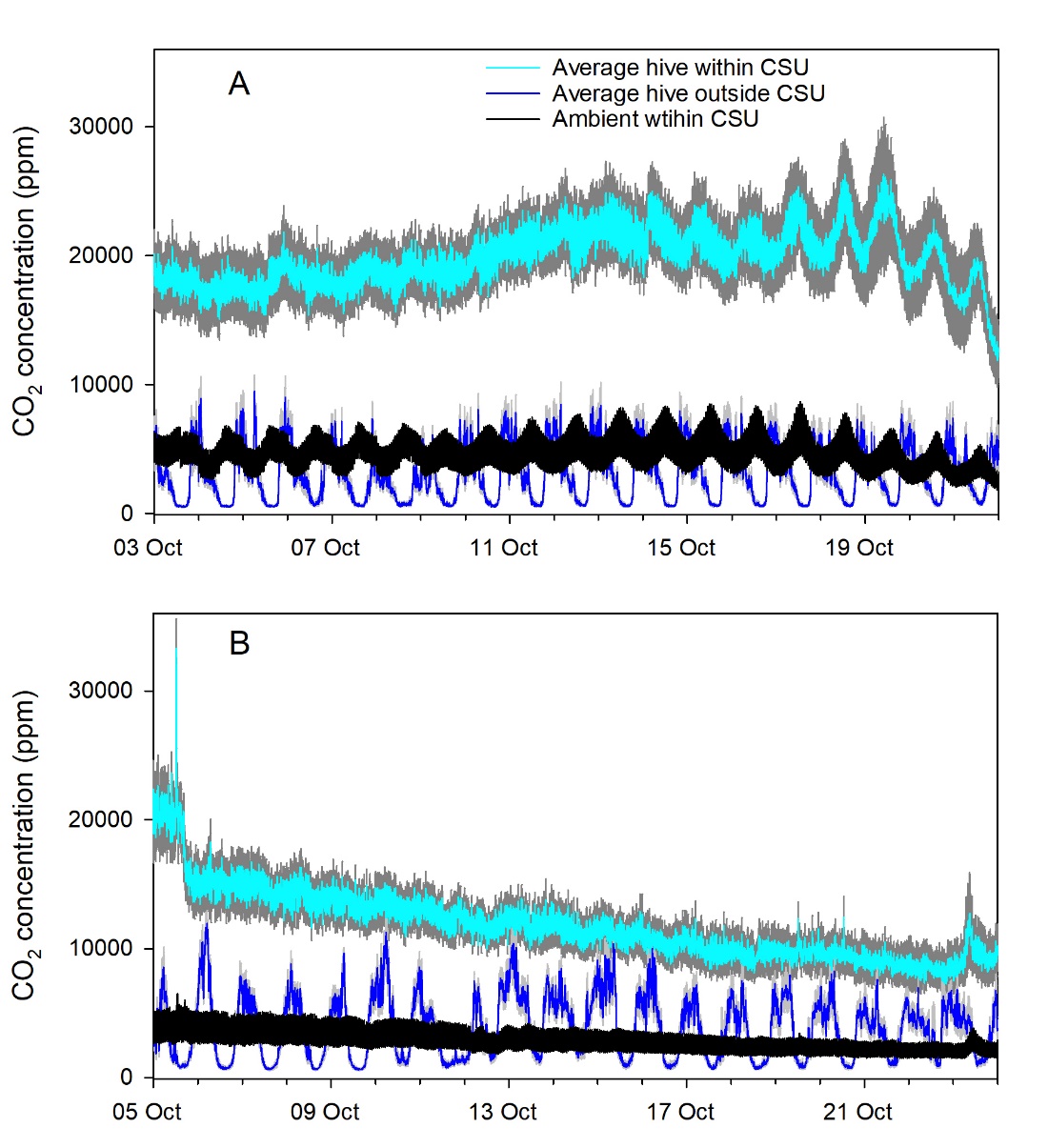


**Figure S3.** Raw CO_2_ concentration data for colonies inside the CSU and outside. A) Fall 2020 experiment; B) Fall 2021 experiment. Standard errors are shown in gray. Hives in the 2020 experiment had migratory entrance screens but those were removed on 5 October in the 2021 experiment. A fan evacuated air for 4 minutes per h in the 2020 experiment and 5 minutes per h in the 2021 experiment.

**
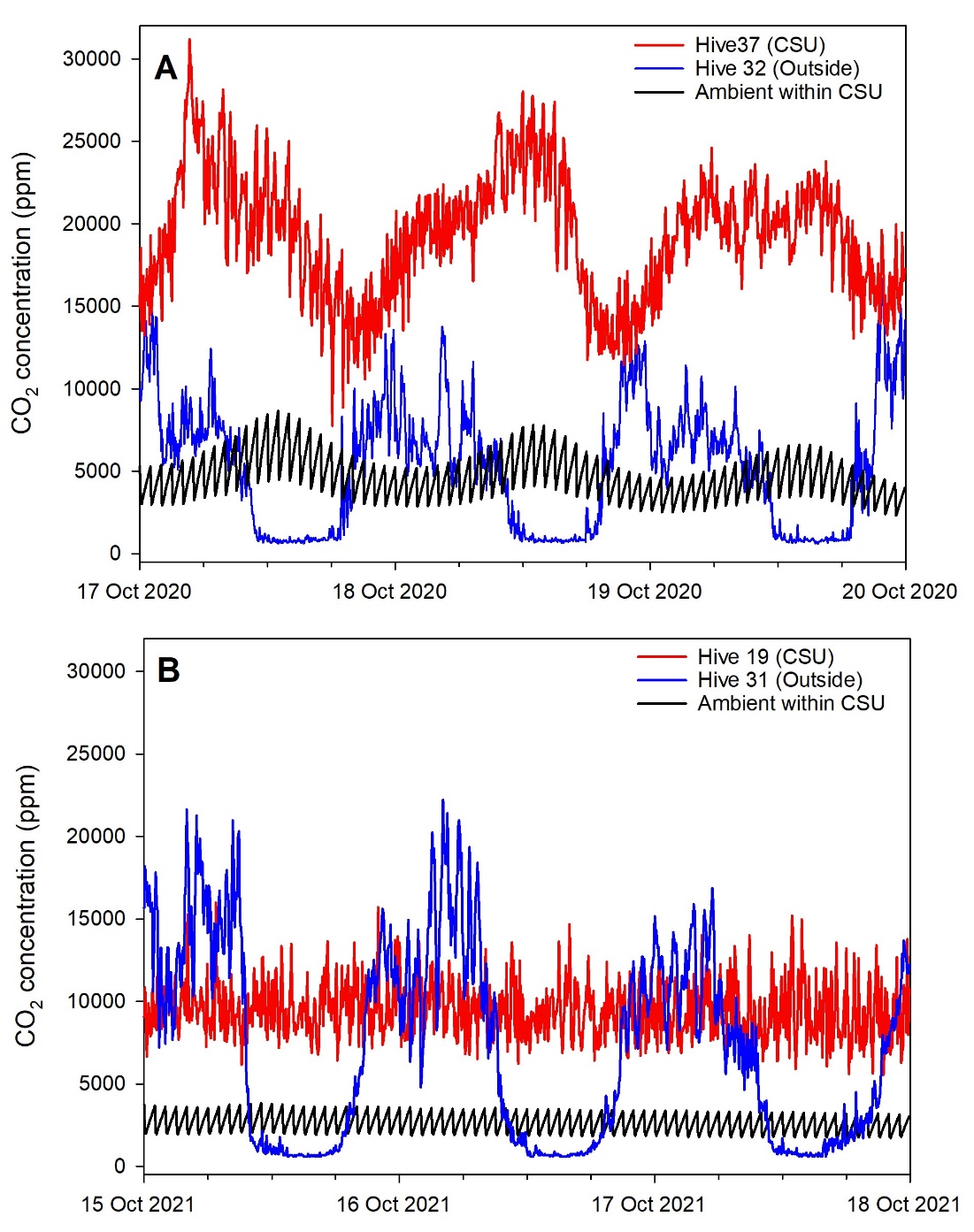
**

**Figure S4.** Sample data for CO_2_ concentrations (every 5 minutes) over 3 d after 2 weeks of storage. A) 2020 experiment; B) 2021 experiment. Average bee colony size in 2021 was more than twice that of colonies in 2020 owing to more favorable bee forage conditions in 2021.


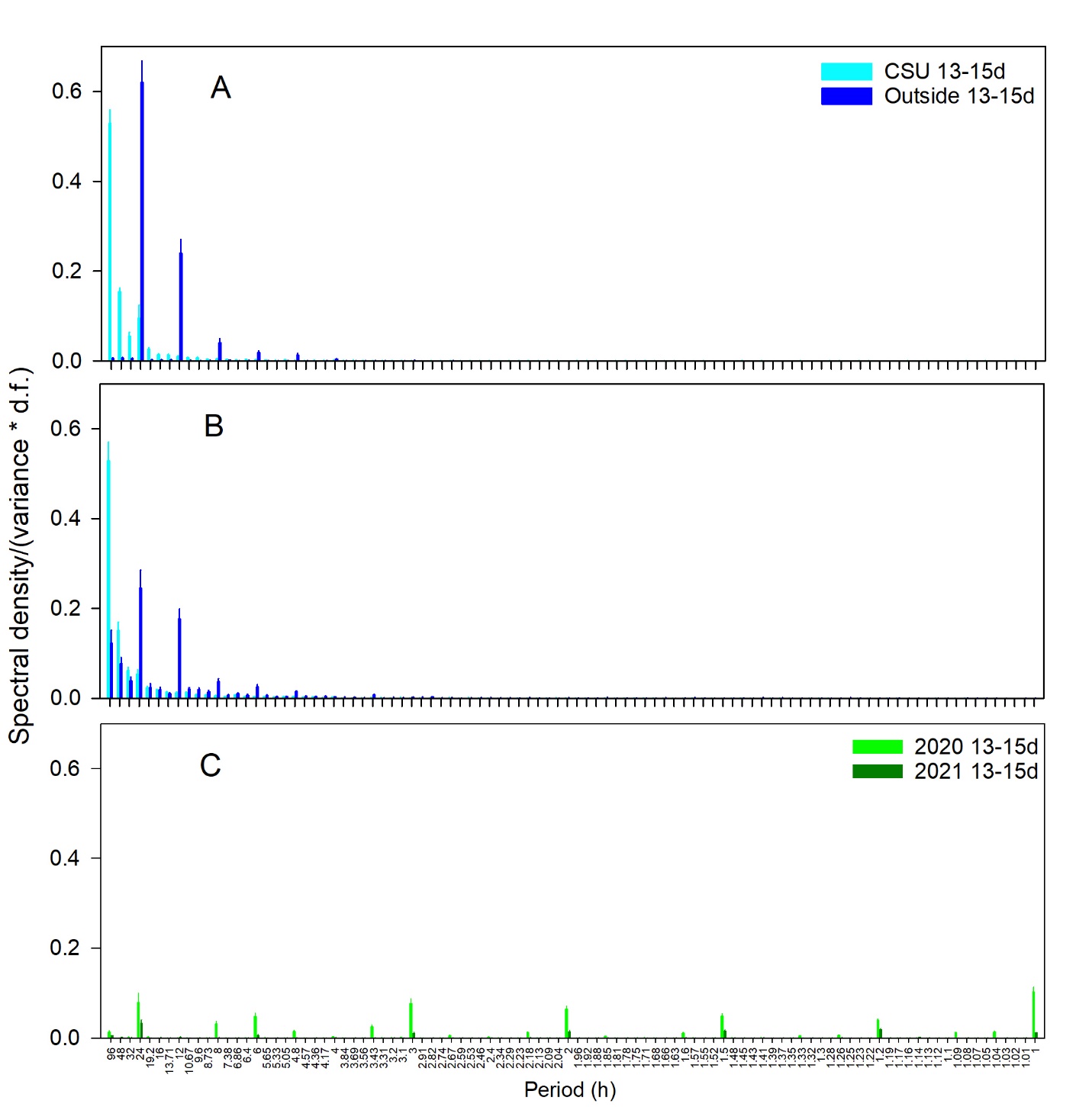


**Figure S5.** Proportion sums of squares (average ± s.e.), calculated as the spectral density divided by the product of the variance and the degrees of freedom, for periods ≥ 1h for raw temperature data. Shown are data for the 5^th^ data subset, from days 13-15. A) 2020 experiment; B) 2021 experiment; C) The same periodogram analysis conducted on the ambient temperature data in the CSU for both years.


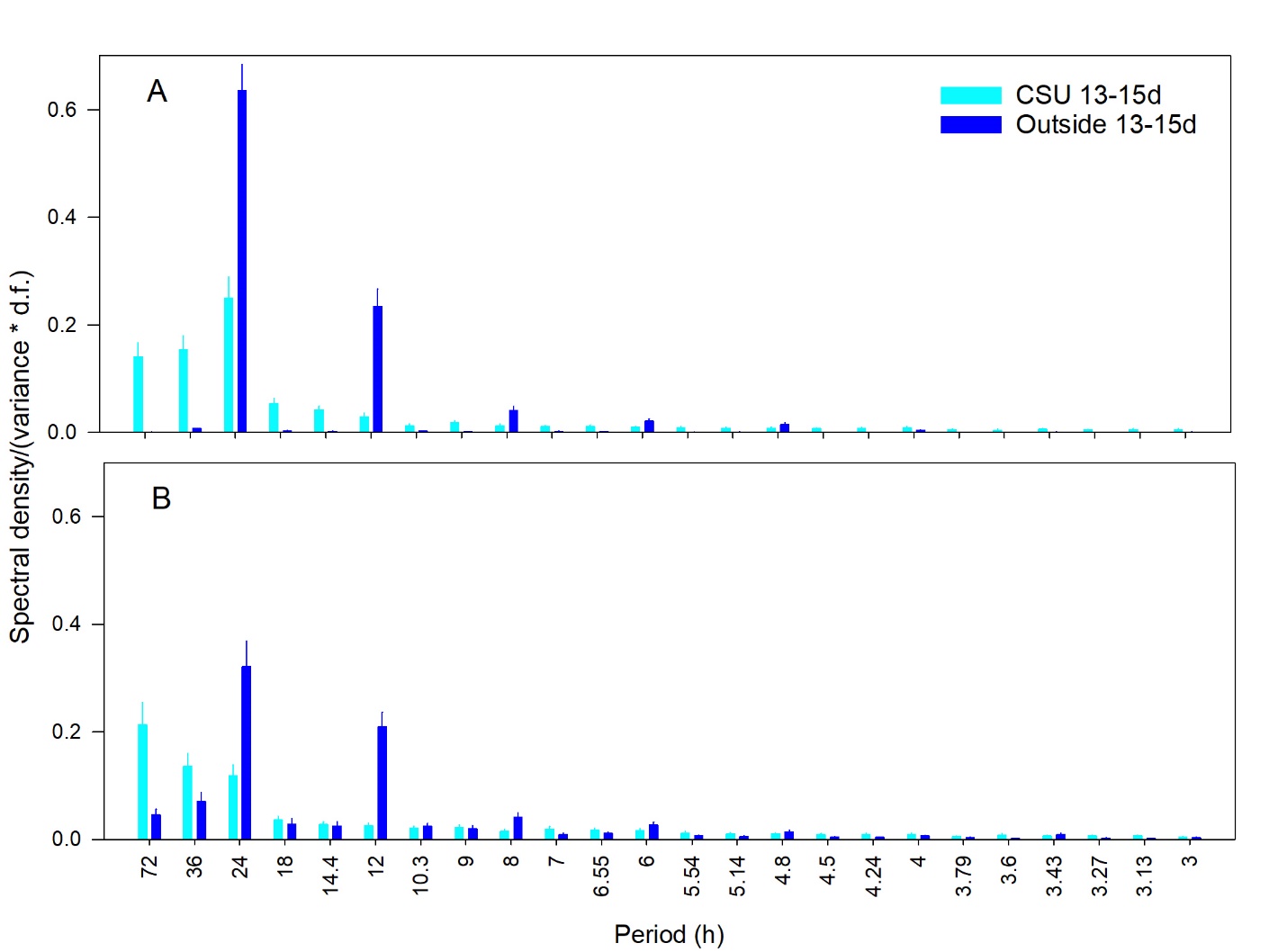


**Figure S6**. Periodograms of the 5^th^ data subset for detrended temperature data (48 h moving average), including only periods of ≥ 3 h. A) 2020 experiment; B) 2021 experiment. Note the spikes for the 72 h period are reduced so the analysis focused periods of 48 h or less. Note also the difference between hives in the cold storage unit and hives kept outside the unit.


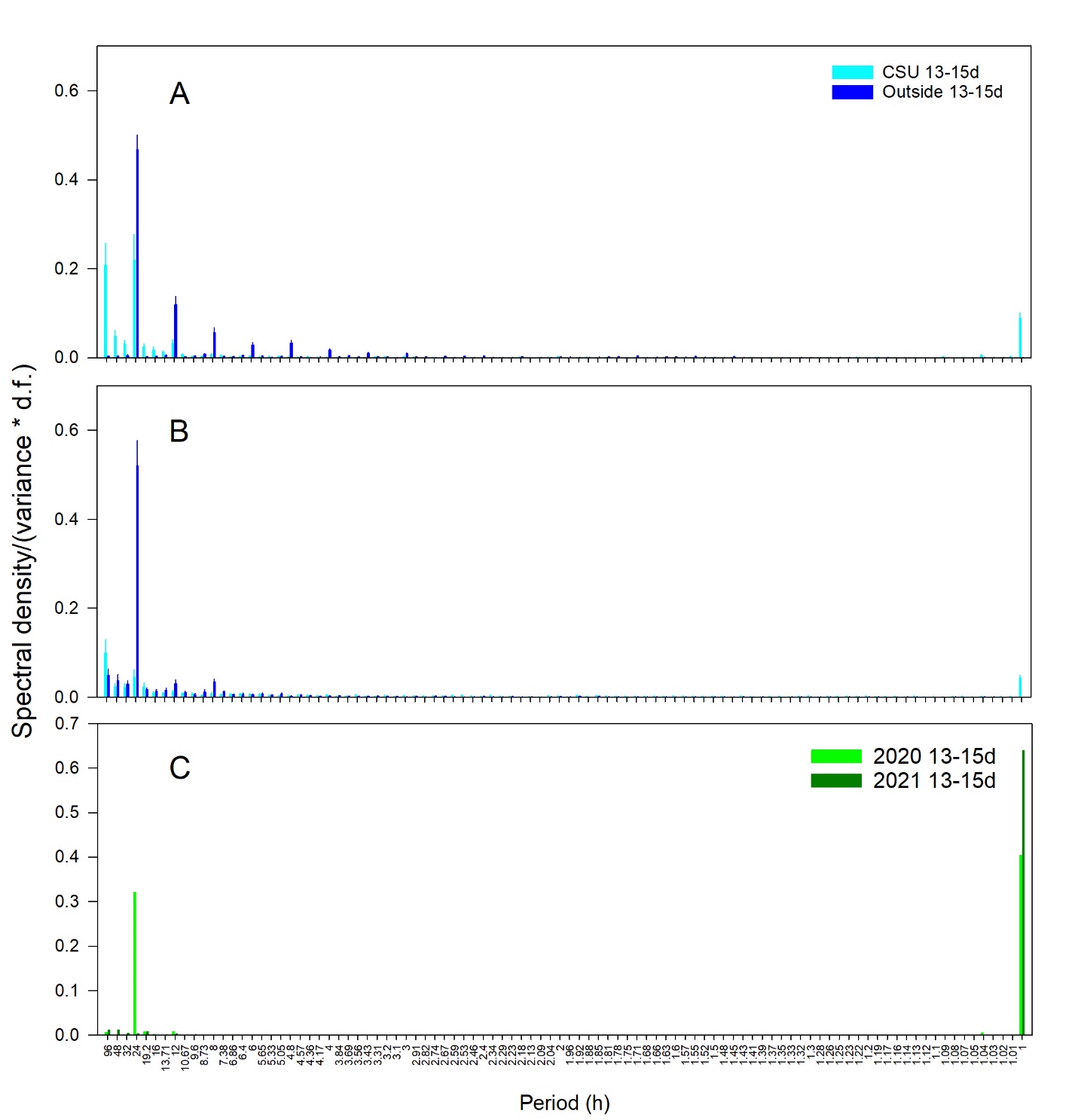


**Figure S7.** Proportion sums of squares (average ± s.e.), calculated as the spectral density divided by the product of the variance and the degrees of freedom, for periods ≥ 1h for raw CO_2_ concentration data. Shown are data for the 5^th^ data subset, from days 13-15. A) 2020 experiment; B) 2021 experiment; C) The same periodogram analysis conducted on the ambient CO_2_ data within the CSU for both years.


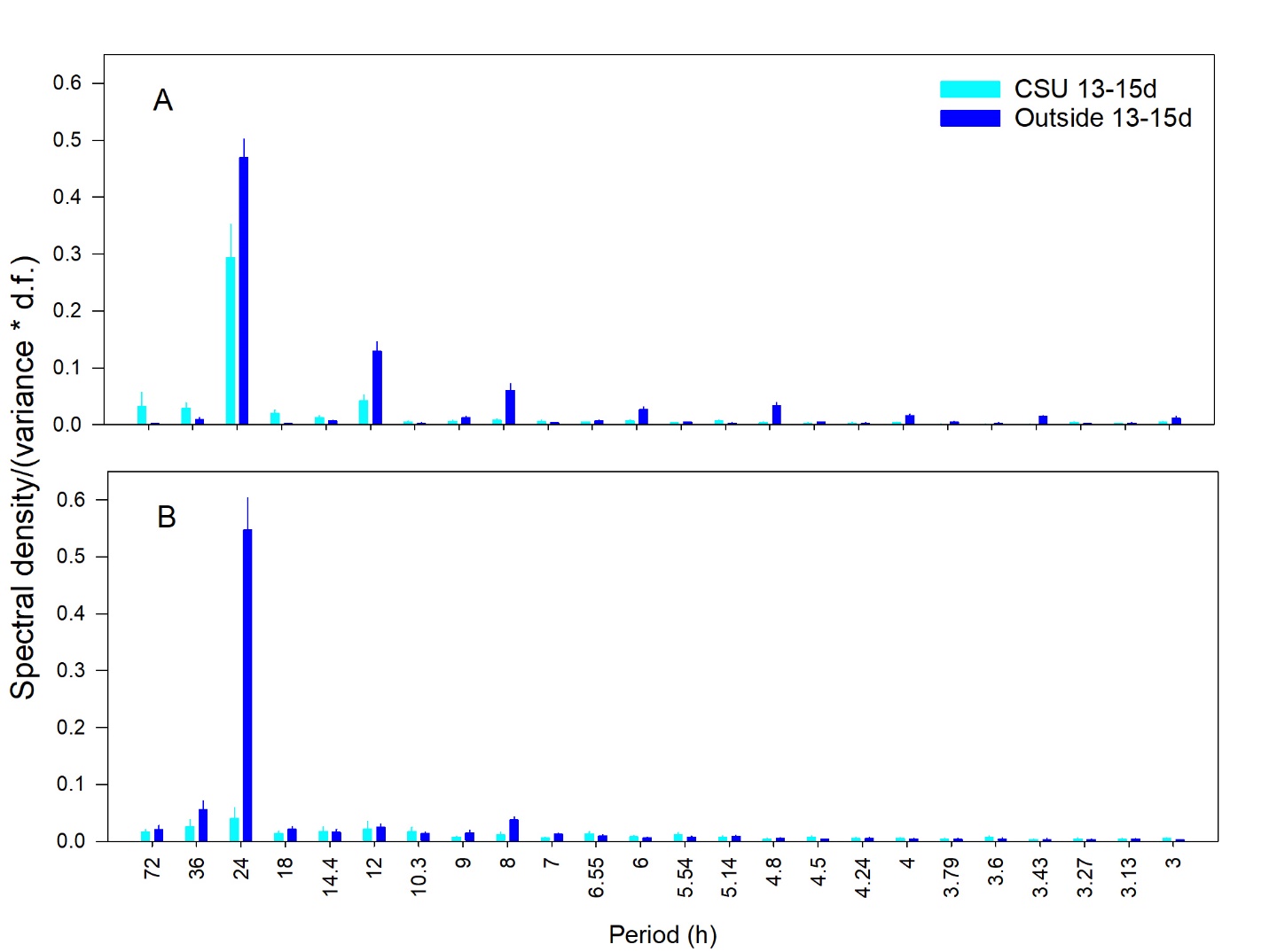


**Figure S8.** Periodograms of the 5^th^ data subset for detrended CO_2_ concentration data (48 h moving average), including only periods of ≥ 3 h. A) 2020 experiment; B) 2021 experiment. Note the spikes for the 72 h period are reduced. This allows the analysis to focus on periods of 48 h or less. Note also the difference between hives in the cold storage unit and hives kept outside the unit.


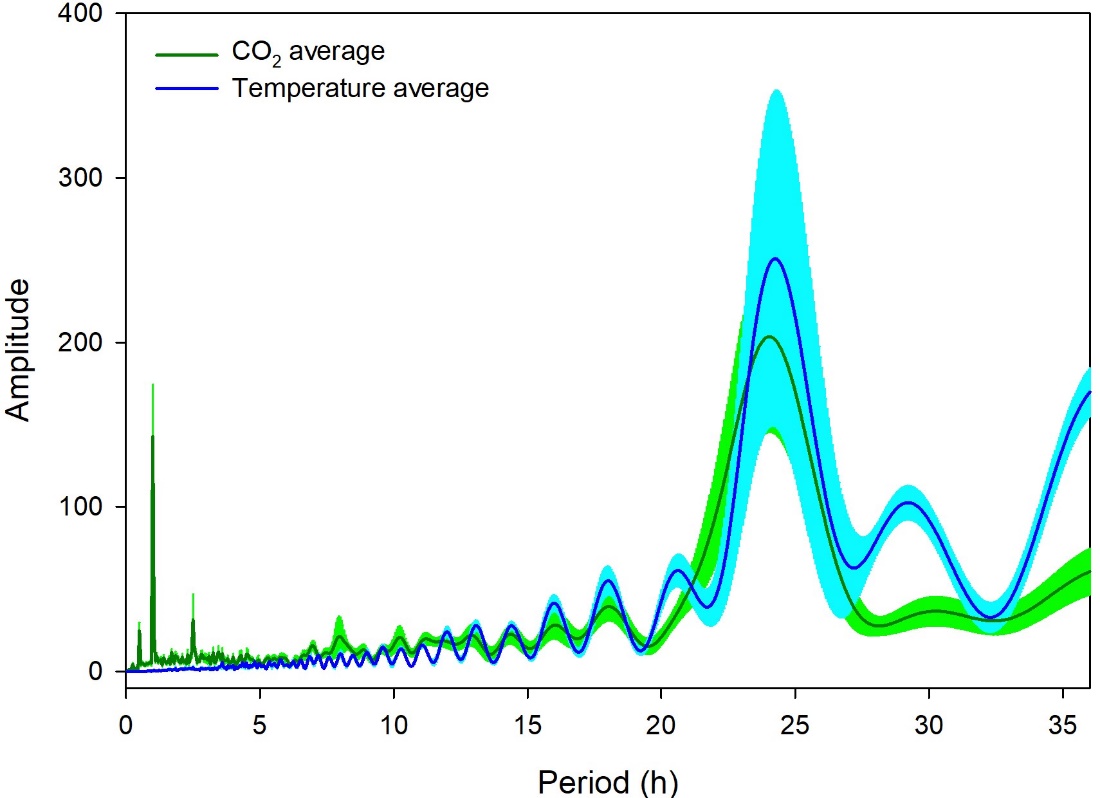


**Figure S9.** Lomb-Scargle periodogram of bee hive temperature and CO_2_ concentration data after 7-12 d total darkness in Winter 2023 and Winter 2024 experiments. Shown are average (solid line) ± s.e. (shading) (N=18). Note the strong 1 h period in the CO_2_ data, owing to the hourly ventilation of the CSU.


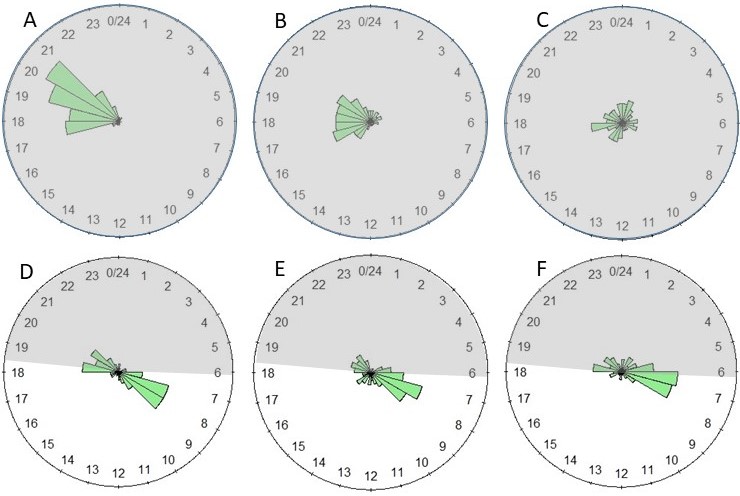


**Figure S10**. Rose diagram of the phase distribution resulting from a cosinor analysis of within-hive detrended temperature data from the Fall 2020 and Fall 2021 experiments. Shown are the phase distribution binned by hour across two 3 d subsets for each graph. Gray shading shows data for colonies kept in the CSU in total darkness. A) Days 1-6 for colonies kept in CSU; B) Days 7-12 for colonies kept in CSU; C) Days 13-18 for colonies kept in CSU; D) Days 1-6 for colonies kept under ambient conditions outside the CSU; E) Days 7-12 for colonies outside the CSU; and F) Days 13-18 for colonies outside the CSU.


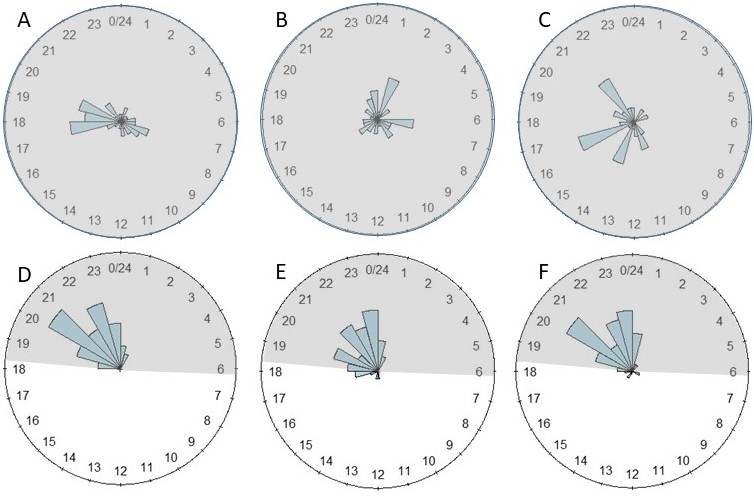


**Figure S11**. Rose diagram of the phase distribution resulting from a cosinor analysis of within-hive detrended CO2 concentration data from the Fall 2020 and Fall 2021 experiments. Shown are the phase distribution binned by hour across two 3 d subsets for each graph. Gray shading shows data for colonies kept in the CSU in total darkness. A) Days 1-6 for colonies kept in CSU; B) Days 7-12 for colonies kept in CSU; C) Days 13-18 for colonies kept in CSU; D) Days 1-6 for colonies kept under ambient conditions outside the CSU; E) Days 7-12 for colonies outside the CSU; and F) Days 13-18 for colonies outside the CSU.


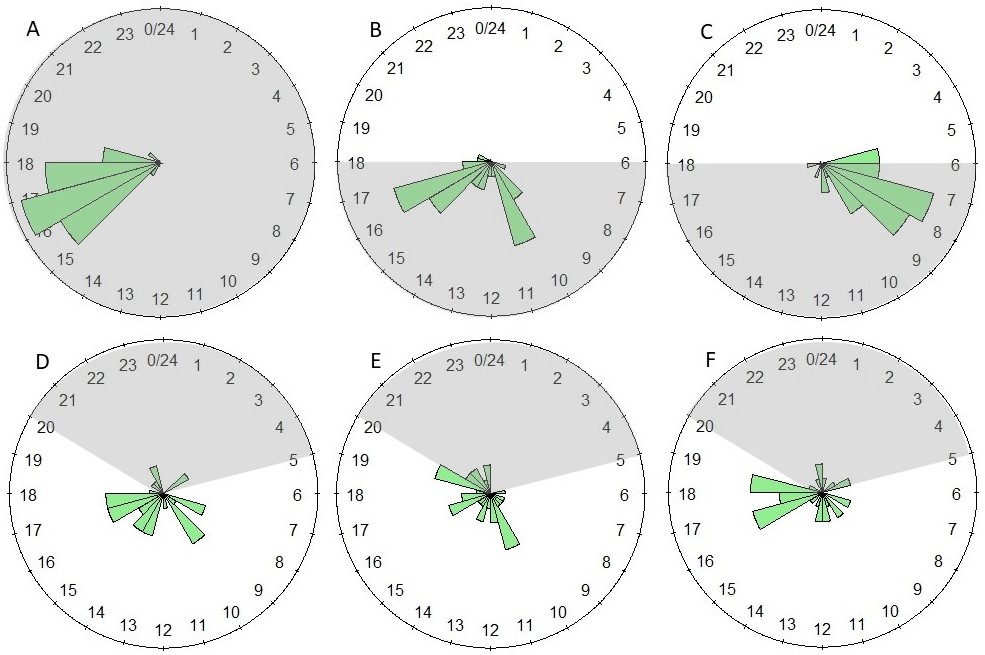


**Figure S12**. Rose diagram of the phase distribution resulting from a cosinor analysis of within-hive detrended temperature data from the Summer 2023 experiment. Shown are the phase distribution binned by hour for 16 hives across two 3 d periods (=32 data points) for each graph. Gray shading shows dark periods (from dusk to dawn for hives outside). A) Days 1-6 for colonies kept in CSU under total darkness; B) Days 7-12 under a 12:12 light:dark regime, with light starting at 6:00 PM and ending at 5:59 AM; C) Days 13-18 under the same light:dark regime as in B; D) Days 1-6 for colonies under ambient conditions outside the CSU; E) Days 7-12 for colonies under ambient conditions; and F) Days 13-18 for colonies under ambient conditions.


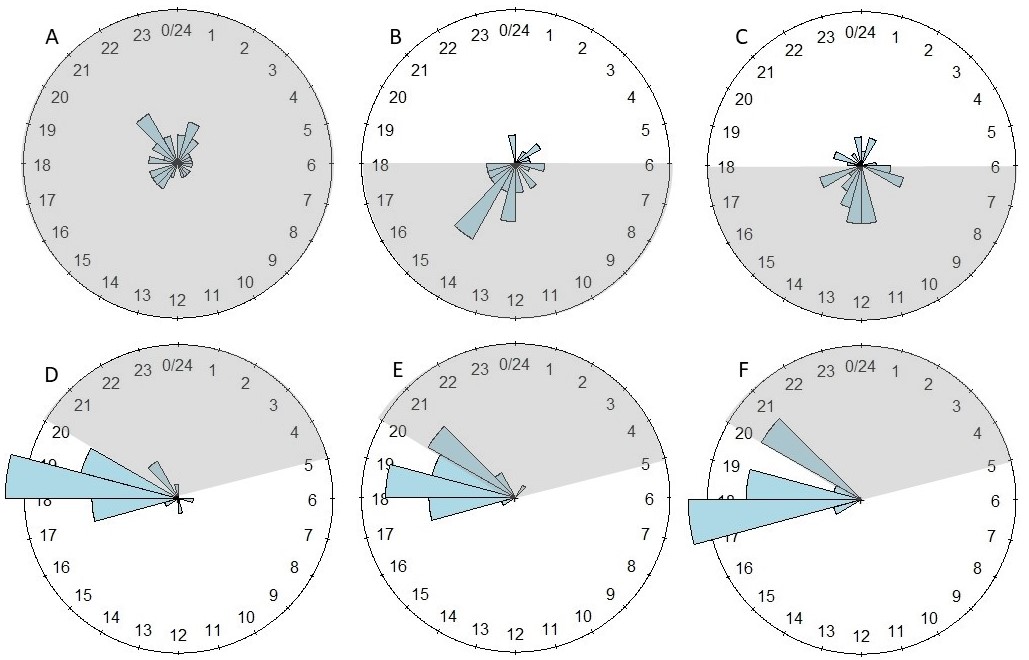


**Figure S13**. Rose diagram of the phase distribution resulting from a cosinor analysis of within-hive detrended CO_2_ data from the Summer 2023 experiment. Shown are the phase distribution binned by hour for 16 hives across two 3-d periods (=32 data points) for each graph. Gray shading shows dark periods (from dusk to dawn for hives outside). A) Days 1-6 for colonies kept in CSU under total darkness; B) Days 7-12 under a 12:12 light: dark regime, with light starting at 6:00 PM and ending at 5:59 AM; C) Days 13-18 under the same light:dark regime as in B; D) Days 1-6 for colonies under ambient conditions outside the CSU; E) Days 7-12 for colonies under ambient conditions; and F) Days 13-18 for colonies under ambient conditions.


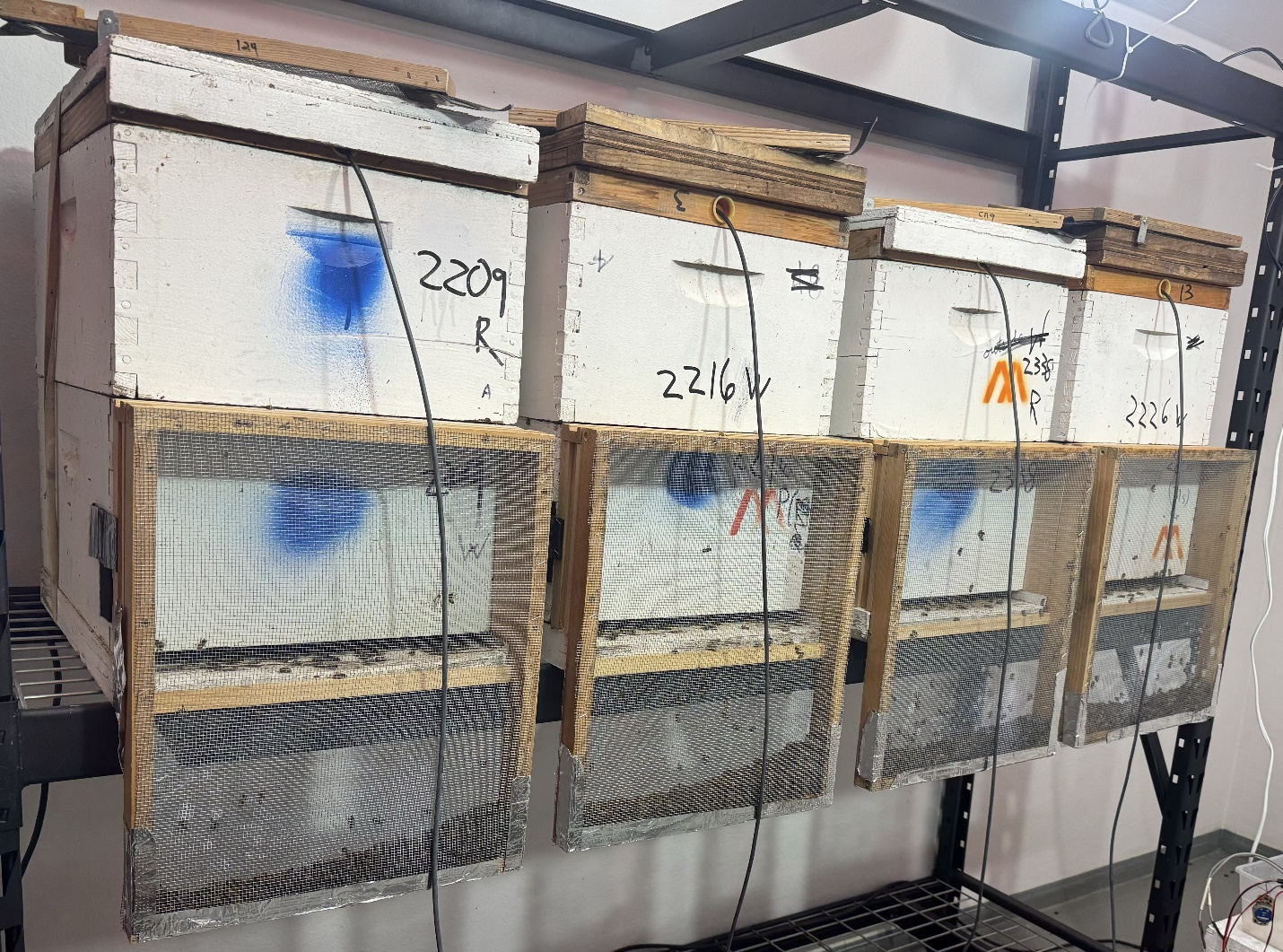


**Figure S14**. Hive entrance covers used in the Cold Storage Unit to keep bees from flying toward the light.
